# Assessing yield stability of pearl millet and rice cropping systems across West Africa using long-term experiments and a modeling approach

**DOI:** 10.1371/journal.pone.0317170

**Published:** 2025-05-27

**Authors:** Louis Kouadio, Kristina Fraser, Ali Ibrahim, Kazuki Saito, Fatondji Dougbedji, Kalimuthu Senthilkumar

**Affiliations:** 1 Africa Rice Center (AfricaRice), Bouake, Côte d′Ivoire; 2 Africa Rice Center (AfricaRice), Regional Station for the Sahel, Saint Louis, Senegal; 3 Faculté d’Agronomie, Université Abdou Moumouni, Niamey, Niger; 4 International Rice Research Institute (IRRI), Metro Manila, Philippines; 5 International Crops Research Institute for the Semi-Arid Tropics (ICRISAT), Bamako, Mali; 6 Africa Rice Center (AfricaRice), Antananarivo, Madagascar; Aberystwyth University, UNITED KINGDOM OF GREAT BRITAIN AND NORTHERN IRELAND

## Abstract

Long-term field experiments (LTEs) provide invaluable insights into temporal yield patterns of agronomic interventions. However, the number of LTEs and agronomic management options tested withing these experiments remain limited compared to the diversity of farming systems in West Africa. Well-tested crop models may be used to identify crop management strategies with high temporal yield stability. This study examines the yield stability of pearl millet and rice under various management options in West Africa, utilizing both experimental and modeling approaches. The Agricultural Production Systems Simulator (APSIM) for pearl millet and rice were calibrated and tested for locally-recommended varieties using LTE data from Niger (pearl millet) and Senegal (rice). Yield stability was evaluated with multiple metrics, including the adjusted coefficient of variation, the sustainable yield index, and the Finlay-Wilkinson regression coefficient. Both APSIM models exhibited a strong performance for grain yield, with Willmott’s indices of agreement at 0.74 for pearl millet and 0.90 for rice, and absolute root mean square errors of 0.19 and 1.20 Mg ha^-1^, respectively. The models effectively reproduced yield stability patterns across a variety of management options including planting date, planting density, fertilizer treatments, and residue retention. Combining fertilizer applications with crop residue retention enhanced yield stability in pearl millet, while season-specific nitrogen management strategies reduced yield variability in rice. Our study underscores the potential of well-tested crop models to complement LTEs in investigating pearl millet and rice yield stability, offering actionable insights for agronomic intensification strategies to enhance productivity and sustainability.

## 1. Introduction

Pearl millet (*Pennisetum glaucum* (L.) R. Br.) and rice (*Oryza* spp.) are important staple food crops in West Africa, contributing substantially to food security and regional economies [1]. The West African region shares approximately 33% of the global millet production, which was estimated to around 30.8 million tons (Mt) in 2023 [2].Rice production was estimated to around 22.2 Mt in 2022, representing ~3% of the global rice production [2]. Top pearl millet and rice producing countries in West Africa include Niger, Nigeria, Mali, Senegal, and Burkina Faso [2]. In these countries the average pearl millet yield is highly variable and often below 800 kg ha^-1^, mainly because of poor soil conditions, variable environmental conditions (e.g., erratic rainfall, wind erosion), and poor or inadequate management systems [3,4]. While pearl millet is largely grown as a subsistence crop in drought-prone semi-arid and arid regions, rice has become an important food crop, favored by continued population growth, urbanization processes with associated dietary changes and economic advancements [5]. As a result domestic rice production across West Africa has gradually increased, from 3.2 Mt in 1980 to 18.5 Mt in 2018, without, however, keeping pace with the domestic demand [6]. Currently, West African rice self-sufficiency rates stand at around 60%, consequently draining the countries’ foreign currency reserves and undermining regional food security [7,8]. Despite the increase in average rice yield from 1.0–1.6 Mg ha^-1^ to 2.1–2.3 Mg ha^-1^ between 1996 and 2018 [9], average rice yields of 4.1, 2.0 and 1.5 Mg ha^-1^ stand against maximum estimated attainable yields of 8.3, 6.5 and 4.0 Mg ha^-1^ in irrigated lowland, rainfed lowland and rainfed upland systems, respectively [1], highlighting existing rice yield gaps. Meanwhile, the Sahelian zone is one of the continent’s most important production areas for irrigated rice because of its largely untapped potential for land expansion and abundant water resources [10], and has thus become a focal area for agricultural intensification efforts [11].

With anticipated changes in rainfall patterns due to a changing climate, coupled with the projected increase in global demand for pearl millet and rice over the next three decades [8,12], addressing climate-related production constraints through agronomic intensification is of paramount interest. Traditional approaches to agronomic intensification have often focused on maximizing productivity [13]. However, the inherent yield variability due to increased climate variability and soil heterogeneity, both from year to year and as a consequence of a changing climate, has largely been overlooked. In fact, climate variability is progressively being associated with a decreased stability of crop yields [14,15], indicating the need for agronomic strategies that exhibit a greater resilience to climatic shocks. Yield stability has hence become a key measure in assessing the resilience of agricultural systems to environmental changes [16]. The assumption is that agricultural systems that respond least to environmental changes are more stable [16]. Recent studies focusing on crop yield stability include the comparison of cropping systems [17], the evaluation of agronomic management practices, e.g., the effects of fertilizers and/or crop residues on yield [4,18,19], and varietal comparisons [20], or effects of climate change on yield stability [21]. The analysis of yield stability typically has relied on the availability of multi-year crop data from long-term field experiments (LTEs) [19,20] or country-level official statistics [21].

Since LTEs are, however, comparatively rare and limited in terms of management options, well-tested crop models can be time- and cost-efficient tools to complement experimental data and to identifying management strategies that maintain temporal yield stability. While crop models have been extensively used to assist in crop agronomy and breeding activities, and to evaluate diverse management options and cropping and farming systems [22,23], only a few studies have reported the use of a crop modeling approach to assess long-term yield stability. For example, the yield stability of rainfed lowland rice systems in two Eastern African countries (Tanzania and Uganda) was quantified using the locally-validated Agricultural Production System Simulator (APSIM)-Oryza model [18]. In their approach, the authors calibrated and validated APSIM-Oryza using a relatively short data period (3-year period) [18,24]. Moreover, given the unavailability of data over longer periods, the capability of the model to replicate the patterns of yield stability found in the experimental data was not evaluated. Likewise, in the case of pearl millet, such comparative study has yet to be reported, highlighting existing knowledge gaps.

In this study, we hypothesized that well-validated crop models using data from LTEs can be used for yield stability assessments of diverse agronomic interventions. Thus, the main objective was to assess the long-term yield stability of various management options in West African pearl millet and rice-based systems using experimental and modeling approaches. More specifically, we aimed at (i) calibrating and validating the APSIM-Millet and APSIM-Oryza models for locally-recommended pearl millet and rice varieties and various management options using LTE data, and (ii) assessing observed and model-estimated yield stability using a range of stability measures.

## 2. Materials and methodology

### 2.1. Experimental sites

This study used data from long-term field experiments (LTEs) from the International Crops Research Institute for the Semi-Arid Tropics (ICRISAT) research station in Sadoré, Niger (13.25°N, 2.283°E, 240 m above sea level) for pearl millet, and the AfricaRice research station in Ndiaye, Senegal (16.219°N, 16.278°W, 12 m above sea level) for rice. The LTE in Niger was established in 2008 to study the effects of fertilizer microdose, crop residue management, and plant density on pearl millet yield and soil fertility [4]. The LTE in Senegal was established in 1991 to investigate the long-term effects of various fertilizer rates on rice yields [25,26].

#### 2.1.1. Environmental conditions.

**Fig 1:**
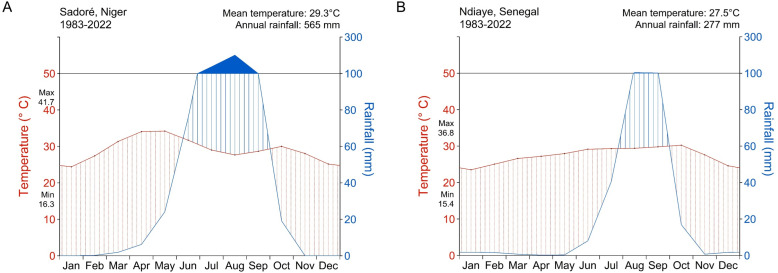
Walter and Lieth climate diagrams. Diagrams for **(A)** Sadoré, Niger and **(B)** Ndiaye, Senegal. The graphs show the monthly averages for temperatures and rainfall, along with annual dry (red dots) and wet (vertical blue lines) periods over a year. Climatic conditions were calculated using data for the period of 1983-2022 for both sites.

The climate in Sadoré is Sudano-Sahelian; it is characterized by a long dry season (November to May) followed by a 5-month rainy season (June-October) (Fig 1A). In Ndiaye the climate is characterized as tropical with a distinct wet season (WS) from July to October, a cold dry season from November to February, and a hot dry season (HDS) from March to June [26]. At both locations, rainfall and temperatures show high interannual variability. In Sadoré for example, average minimum and maximum daytime air temperatures between 1983 and 2022 were around 16 °C and 42 °C, respectively, with monthly average rainfall during the rainy season of about 143 mm and annual rainfall of 565 mm over the 40-year period (Fig 1A). In Ndiaye, the annual rainfall ranged from 100 and 620 mm (mean of 277 mm) between 1983 and 2022, with > 80% occurring between July and October (Fig 1B). Minimum daytime temperatures were below 20 °C during November to March, while maximum daytime temperatures were above 35 °C from April to June over the 40-year period.

The soil in Sadoré is classified as sandy, siliceous, isohyperthermic psammentic Paleustalf [27] and as a typical orthithionic Gleysol, according to the FAO systematic, in Ndiaye [26]. The Gleysol is of fluviatile origin underlain by marine sand deposits and generally fine-textured [25].

#### 2.1.2. Experimental designs.

At Sadoré, LTEs were established in 2008 to investigate the effect of fertilizer microdose with crop residue (CR) removal on pearl millet yield [4]. They were designed as a 3 × 2 factorial experiment– three levels of fertilization × two plant densities– organized in randomized complete block design with three replications (individual plot size of 7 m × 7 m). The same design was adopted in 2011 for another experiment aiming at exploring the effect of CR retention [4]. In this study we used data for the period of 2011–2018. All experiments were conducted under rainfed conditions and involved cultivar Haïni Kirey Précoce (HKP). The two plant densities tested were 10,000 pockets ha^–1^ (PDENS1), which is the official recommendation [4], and 15,000 pockets ha^–1^ (PDENS2). Plants were thinned at two per pocket two weeks after sowing. Fertilizer treatments included a control plot (without fertilizer) and two microdose fertilizer treatments: (i) 2 g diammonium phosphate (DAP) fertilizer per planting hole, and (ii) 6 g of 15-15-15 NPK fertilizer per planting hole (Table 1). Pearl millet residues from the previous harvest were applied at the start of each season at a rate of 0.8 Mg ha^-1^ in control plots and 1.4 Mg ha^-1^ in microdose fertilizer plots. Each fertilizer and crop residues treatments were applied to the same plot over the study period. Weed, pest and disease management were conducted as needed to keep the plots weed and disease-free. More details on the experimental settings can be found in [4].

**Table 1:** Overview of agronomic details of the pearl millet experiments at Sadoré, Niger during the period of 2011–2018. The variety grown was Haïni Kirey Précoce (HKP).

Sowing dates and plant density	Fertilizer rates	Residue management
Sowing dates:	T0 (control): 0 kg ha^-1^	Residue removal
20 June 2011, 12 June 2012, 2 July 2013, 1 June 2014, 9 July 2015, 15 June 2016, 14 June 2017, 24 June 2018	T1: 2 g DAP + 1 g Urea. ↔ PDENS1: 8.2 kg N ha^-1^ + 4.02 kg P ha^-1^ ↔ PDENS2: 9 kg N ha^-1^ + 3.93 kg P ha^-1^ + 7.47 kg K ha^-1^	Residue incorporation: 0.8 Mg ha^-1^ in T0 plots 1.4 Mg ha^-1^ in fertilizer microdose plots
Plant density: PDENS1: 10,000 pockets ha^-1^ at 2 plants per pocket; that is, 2 plants m^-2^ PDENS2: 15,000 pockets ha^-1^ at 2 plants per pocket; that is, 3 plants m^-2^	T2: 6 g NPK (15-15-15) ↔ PDENS1: 12.3 kg N ha^-1^ + 6.03 kg P ha^-1^ ↔ PDENS2: 13.5 kg N ha-1 + 5.9 kg P ha^-1^ + 11.21 kg K ha^-1^	

At Ndiaye, experiments were established as a continuous double cropping system during the WS and HDS as a randomized complete block design with a total of six treatments and four replications each. Four fertilizer treatments were relevant for this study: (i) 0–0–0 kg NPK ha^-1^, (ii) 60-26-50 kg NPK ha^-1^, (iii) 120-26-50 kg NPK ha^-1^, and (iv) 180-26-50 kg NPK ha^-1^. PK was applied basally 14 days after transplanting, and N was applied at rates of 50:25:25 at 14 days after transplanting, panicle initiation and at around 10 days before flowering, respectively (Table 2). Individual plots of 5 m × 5 m were levelled and puddled prior to rice sowing, and each fertilizer treatment was applied to the same plot over time. Rice nurseries were established in February (42–48 day of the year (DOY)) for the HDS and in July for the WS (196–208 DOY) and transplanted after 19–31 days at 25 hills m^-2^. The short duration variety Sahel 108 (IR 13240–108-2-2-3) was used. All treatments were fully irrigated from transplanting until 14 days prior to maturity and aboveground residues were removed from the field after rice harvest. Weeds were controlled manually as needed to keep the plots weed-free. Standard pest and disease management practices were used. More details can be found in [26].

**Table 2:** Overview of agronomic details of the rice experiments at Ndiaye, Senegal during the period of 2015–2022. The variety grown was *Sahel 108.*

Variety selection and establishment	Urea-N rates	Water management
Transplanted [SBDUR 19-31, 2 seedlings/hill, 25 hills m^-2^]	0, 60, 120, and 180 kg ha^-1^ Split-application 50:25:25 [14 DAT, panicle initiation and 10 days prior flowering]	Fully irrigated from planting until 2 weeks prior maturity; triggered if pond depth < 5 cm]
Rice sowing HDS [DOY 42–48] WS [DOY 196–208]		

SBDUR, seedbed duration; HDS, hot dry season; WS, wet season; DOY, day of year; DAT, days after transplanting.

#### 2.1.3. Experimental data.

For pearl millet, experimental data for the period of 2011–2018 including aboveground biomass and yield data, and management practices (i.e., sowing and harvest dates, plant density, organic and inorganic fertilizer rates and dates of applications) were used in this study. Daily records of rainfall, minimum temperature, maximum temperature, and solar radiation for the 1983–2022 period were obtained from the climate station at the ICRISAT experimental site. Gaps in the climate data were filled using gridded data for the study site retrieved from the NASA POWER data portal (https://power.larc.nasa.gov/). Soil data were from [4,28,29]. Whilst detailed information was available for the top layers (0–20 cm), data were unavailable for the remaining layers (Table 3). To fill the gaps, relevant information from the International Food Policy Research Institute’s HarvestChoice Dataverse [30] or as recommended for the soil module in APSIM [31] were used (Tables 3 and 4).

**Table 3:** General soil physical and chemical properties of the Arenosol in Sadoré, Niger (determined in 2008) and the Gleysol in Ndiaye, Senegal (determined in 1997).

Site	Depth	BD	Clay	Sand	Texture	SOM	C_org_	N_tot_	NO_3_	NH_4_^+^	C:N ratio
	[cm]	[g cm^-3^]	[%]	[%]	[WRB]	[g kg^-1^] ^a^	[g kg^-1^] ^a^	[g kg^-1^]^a^	[ppm]	[ppm]	[-]^a^
**Sadoré, Niger**	5-10	1.55	9.1	85.5	Loamy sand	–	2.6	0.2	0.600	0.400	12.1
	10-20	1.54	9.1	85.5	Loamy sand	–	2.6	0.2	0.600	0.400	12.1
	20-30	1.48	6.0	90.0	Sand	–	–	–	0.300	0.300	–
	30-40	1.59	6.0	90.0	Sand	–	–	–	0.100	0.300	–
	40-50	1.59	7.0	89.0	Sand	–	–	–	0.100	0.063	–
	50-70	1.58	8.0	88.0	Loamy sand	–	–	–	0.100	0.032	–
	70-100	1.62	7.0	88.0	Loamy sand	–	–	–	0.100	0.021	–
	100-150	1.61	7.0	86.0	Loamy sand	–	–	–	0.100	0.006	–
**Ndiaye, Senegal**	0-21	1.50	40.0	16.0	Silty clay	16.0	9.3	1.0	–	–	9.3
	21-53	1.59	54.0	16.0	Clay	7.4	4.3	0.3	–	–	14.3
	53-73	1.38	69.0	10.0	Clay	5.5	3.2	0.1	–	–	32.0
	73-100	1.58	35.0	54.0	Sandy clay	3.4	2.0	0.1	–	–	20.0

BD, bulk density; soil texture definition according to the World Reference Base for Soil Resources (WRB): sand (63–2000 µm), silt (2–63 µm), clay (< 2 µm); SOM, soil organic matter content; C_org_, soil organic carbon content; N_tot_, total soil N content; NO_3_^-^, soil nitrate content; NH_4_^+^, soil ammonium content.

^a^For pearl millet, the values measured for C_org_, N_tot_ and C:N ratio were only available for the 0–20 cm soil layers. No measurements were carried out for SOM.

**Table 4:** Soil properties of the Arenosol in Sadoré, Niger, and the Gleysol in Ndiaye, Senegal used for APSIM modeling.

Site	Depth [cm]	AirDry [cm^3^ cm^-3^]	LL15 [cm^3^ cm^-3^]	DUL [cm^3^ cm^-3^]	SAT [cm^3^ cm^-3^]	KS [mm day^-1^]	SWCON [0-1]	*fBiom* [0-1]	*fInert* [0-1]
**Sadoré, Niger**	5-10	0.015	0.030	0.070	0.370	–	0.410	0.030	0.400
	10-20	0.020	0.030	0.070	0.370	–	0.410	0.030	0.400
	20-30	0.020	0.030	0.070	0.390	–	0.410	0.020	0.700
	30-40	0.040	0.040	0.070	0.350	–	0.410	0.015	0.750
	40-50	0.040	0.040	0.070	0.350	–	0.410	0.010	0.800
	50-70	0.040	0.040	0.070	0.350	–	0.410	0.010	0.950
	70-100	0.040	0.040	0.070	0.340	–	0.410	0.010	0.950
	100-150	0.040	0.040	0.070	0.340	–	0.410	0.010	0.950
**Ndiaye, Senegal**	0-21	0.120	0.243	0.376	0.433	11.76	0.500	0.040	0.650
	21-53	0.254	0.317	0.414	0.419	0.00	0.300	0.030	0.750
	53-73	0.316	0.316	0.434	0.478	5.28	0.300	0.010	0.930
	73-100	0.211	0.211	0.314	0.406	47.52	0.700	0.010	0.930

Layer-wise volumetric water content at air dry (AirDry), wilting point (LL15), field capacity (DUL) and saturation (SAT); KS, saturated percolation rates; SWCON, soil water conductivity; initial labile (*fBiom*) and inert (*fInert*) fraction of soil organic carbon.

At Ndiaye, a total of 15 consecutive rice growing seasons (eight HDS, seven WS) from 2015 to 2022 and four fertilizer treatments (0, 60, 120 and 180 kg N ha^-1^) were used. Daily rainfall, maximum and minimum temperatures, and solar radiation from 1980–2022 were recorded by an automatic weather station at the Africa Rice Center research station (16.23°N, 16.23°W). Missing data were filled with locally-reconstructed historic daily climate data (1995–2014) [32] and data obtained from the NASA POWER data portal. Soil characteristics were determined in 1997 from soil profiles adjacent to the trials [25]. Topsoil soil organic carbon content (C_org_) was 9.3 g kg^-1^ and subsequently decreased to 2 g kg^-1^ at 73–100 cm depth (Table 3). Similarly, the topsoil N content (N_tot_) was low with 1 g kg^-1^ and subsequently decreased to 0.1 g kg^-1^ and was thus below the critical N content for rice growth of 2 g kg^-1^ [33] (Table 3). The experimental data collection for the 2015–2022 period included phenological key stages, i.e., sowing, transplanting, panicle initiation, flowering, physiological maturity, final biomass and grain yields, as well as details on fertilizer application dates.

### 2.2. The APSIM-Millet and APSIM-Oryza models

APSIM is a modular simulation framework that operates on a daily time-step and at point scale, allowing the versatile specification of management options and simulation of agricultural system performances [34]. In this study, the APSIM-Millet and APSIM-Oryza models (v. 7.10) were used for pearl millet and rice growth and yield simulations, respectively. For pearl millet simulations, the SurfaceOM, SoilWat, SoilN modules, and a recently developed pearl millet module [35] were used. This latter module integrates recent advancements in modeling of the tillering mechanism in sorghum [36,37] to improve the growth and development processes of pearl millet. The generic functions used in APSIM-Millet can be found at https://www.apsim.info/documentation/model-documentation/crop-module-documentation/millet/.

For rice growth modeling, the Oryza, SurfaceOM, SoilWat, SoilN and Pond modules were used. APSIM-Oryza integrates the rice physiological routines of ORYZA2000 [38] to simulate the growth and development of rice, the water- and N-uptake as well as abiotic stresses and the responses thereof [39]. However, APSIM-Oryza uses APSIM soil routines [40,41]. For temporarily and/or permanently flooded soil conditions, both SoilWat and SoilN were modified [40], as well as the Pond module developed to simulate key chemical and biological processes under submerged conditions [41].

#### 2.2.1. Parameterization and calibration of APSIM models.

Model parameterization refers to the process of supplying the model with measured and/or derived input parameters and variables such as daily climate data, soil physical and chemical characteristics. Climate data include minimum and maximum temperatures, solar radiation and rainfall. Soil data include layer-wise pH, bulk density, soil volumetric water contents at saturation, field capacity, permanent wilting point, and initial soil organic carbon (C_org_) and mineral nitrogen (NO_3_^-^, NH_4_^+^) contents. Information on crop management practices, including sowing/transplanting dates, plant density, sowing depths, harvest dates, fertilizer rates and application dates, and irrigation management, is also needed for APSIM simulations. Not directly measurable parameters such as those governing phenology, biomass partitioning, and soil mineralization capacities require iterative calibration and/or adjustment within plausible bounds.

##### Soil organic matter mineralization.

For soil organic matter (SOM) mineralization processes, APSIM requires parameter values for *fBiom* (fraction of soil microbial biomass) and *fInert* (fraction of inert humic material in SOM) to initialize the proportions of SOM pools and their mineralization capacities. For pearl millet modeling, there was no calibration for *fBiom* and *fInert* because of the unavailability of relevant data. Therefore we used the values reported in [29] for soil layers up to 40-cm depth, along with the default values recommended in the literature for soil layers beyond 40-cm depth [31] (Table 4). For rice modeling, *fBiom* and *fInert* were calibrated using yield data from the 0–0–0 kg NPK ha^-1^ treatments. Parameter values were incrementally adjusted within physically plausible bounds [42] and until the predicted indigenous N supply in the 0–0–0 kg NPK ha^-1^ treatments allowed close simulation of observed rice yields (Table 4).

##### Soil water conductivity.

The macro flow conductivity and the vertical water flow through macropores is specified by measured saturated percolation rates, while soil water conductivity, i.e., the proportion of water exceeding DUL and draining daily into the subsequent soil layer, is specified via the *swcon* coefficient. The s*wcon* coefficient varies depending on soil texture; it is typically below 0.5 for heavy clay and above 0.8 for sandy soils given their higher hydraulic conductivity [42].

##### Pearl millet phenology and assimilate partitioning.

For APSIM-Millet calibration, the parameters tt_emerg_to_endjuv (thermal time from emergence to end of juvenile phase), tt_endjuv_to_init (thermal time from end of juvenile to floral initiation), tt_flag_to_flower (thermal time from flag leaf emergence to flowering), tt_flower_to_start_grain (thermal time from flowering to start of grain filling)), and tt_flower_to_maturity (thermal time from flowering to maturity) were adjusted through a try-error process based on the reported harvest dates during the study period and the 60–65 days after sowing window reported in the literature for the flowering stage for the variety HKP [43]. The parameters tt_endjuv_to_init, dm_per_seed (dry matter content per seed), maxGFRate (maximum grain filling rate), aX0 (largest leaf multiplier), aMaxS (largest leaf area factor), and aMaxI (intercept for largest leaf calculation) were adjusted using the function ‘optim_apsim’ of the package ‘apsimx’ [44]. Through the ‘optim_apsim’ function, mean errors between observed and predicted yields were minimized and the best combination of crop parameters was returned. Moreover, based on the range of radiation use efficiency values reported in [29], the default radiation use efficiency value (1.86) was varied between 1.30 and 1.86 by step of 0.1 for potential model improvement.

Crop parameters values from the literature [28,29] and parameter values for the default cultivars in the standard version of the model were used to define the initial parameter values and to check the plausible range within which each parameter can vary. Note that the impacts on yield of pests and diseases are not simulated in the model. Likewise, given that crop response to potassium (K) fertilizer is not simulated in APSIM, the K rate in each of the fertilizer microdose treatments in pearl millet experiments (Table 1) were not considered in the simulation configurations.

##### Rice phenology, assimilate partitioning, and floret sterility.

APSIM uses growing degree days (GDD; °C d^-1^) to determine the phenological development of rice [45]. Genotype-specific GDD constants and partitioning coefficients were calibrated from observed key phenological stages and corresponding biomass data. Cultivar-specific temperature sums (GDD) needed to complete each development stage were determined using the Blackman equation (Eq. (1)) [46]:


$$\text{T}{\text{I}}_{\text{t}}=\max(0,\;{\text{T}}_{\text{t}}-\text{TBD}textforT_{t}<\text{TOD}$$ (1)

where TI_t_ (°C) is the increment in thermal time over time unit *t*, T_t_ is the average ambient temperature during *t*, TBD is the base temperature and TOD the optimal temperature for development. A TBD of 15 °C and TOD of 34 °C were used following [46]. Similarly, field data was used to determine leaf maximum and minimum relative growth rates, maximum individual grain weight at maturity, and specific leaf area. Results were further fine-tuned to match the observed data best.

Following the ORYZA2000 model [45], APSIM-Oryza uses ambient air temperature to calculate floret sterility from high temperatures during the flowering period (0.96 ≤ DVS ≤ 1.22). Thus, average maximum ambient air temperatures of above 36.6 °C cause proportional floret sterility [45]; this threshold is then used as a default input parameter (httmax = 36.6 °C). However, it is known that the developing floret is exposed to the canopy temperature rather than the ambient temperature [47], which can be as much as 6–7 °C lower in dry environments. At Ndiaye studies reported that in rice cv. Sahel 108 the canopy temperature was about 4 °C lower than ambient air temperature during flowering [48]. Therefore, and to avoid the overestimation of floret sterility, the httmax of 36.6 °C was increased to 40.6 °C (i.e., increasing the threshold that is inducing floret sterility).

#### 2.2.2. Validation of APSIM models.

For APSIM-Millet calibration and validation, we used the data from fertilizer treated plots under crop residues retention (CR_ret_). This dataset was split in two based on the fertilizer treatment: the calibration data set consisted of all data for T2 under PDENS2, whereas the validation data set included the remainder of the data under CR_ret_ (T2-PDENS1, T0 and T1 for both PDENS1 and PDENS2). We also carried out a second model evaluation by using all the data under CR removal (CR_rmv_). For this latter case, the amount of initial CR in the model simulation configurations was set to 0 for control and fertilizer treated plots.

For APSIM-Oryza, field data from 2016, including the HDS and WS seasons, were used for model parameterization and calibration. Default crop values (rice variety IR 72) for phenological development and biomass partitioning were adjusted using the phenological and yield data from the 180 kg N ha^-1^ treatment, and until observed and predicted data provided a good fit. The remaining field data from seven HDS and six WS between 2015–2022 were subsequently used for model validation.

#### 2.2.3. Sensitivity analysis.

The Gaussian Emulation Machine for Sensitivity Analysis (GEM-SA) software package [49,50] was employed to investigate the sensitivity of simulated yield and biomass at harvest to selected crop parameters. For pearl millet, 18 cultivar-specific parameters and the radiation use efficiency (RUE) were evaluated in the sensitivity analysis (S1 Table). RUE was included due to its potential to influence biomass and yield simulations in APSIM-Millet [29]. For rice, seven crop parameters were evaluated (Table 5). The LP-tau method was used to generate a design matrix of 400 parameter combinations based on the parameter ranges for each crop (Table 5). Each parameter combination was then run using the relevant crop model. For pearl millet, APSIM-Millet was run for the period of 2011–2018 using the model configuration for Sadoré with plant density PDENS2 and fertilizer treatment T2 under CR retention (Table 1). For rice, APSIM-Oryza was run for the period of 2015–2022 using the model configuration for Ndiaye with fertilizer rate = 180 kg N ha^-1^ for both cropping seasons each year (Table 2). Note that some parameter combinations did not produce an output due to either crop failure or the crop not reaching maturity by the end of the simulation period. These parameter combinations were discarded from the analysis.

**Table 5:** Parameters and ranges of values used in sensitivity analysis of simulated pearl millet and rice yield and biomass.

Crop	Parameter	Acronym	Unit	Range
**Pearl millet**	Intercept for largest leaf calculation	aMaxI	(-)	-50–50
	Largest leaf Area Factor	aMaxS	(-)	1–20
	Largest leaf multiplier	aX0	(-)	0.5–0.9
	Dry matter content per seed	dm_per_seed	(g)	0.0001–0.001
	Power coefficient for TPLAmax = TLN^coef	main_stem_coef	(°C^-1^)	1–3.5
	Maximum grain filling rate	maxGFRate	(mg °C d^-1^)	0.001–0.05
	Radiation use efficiency	rue	g MJ^-1^	1.3–2.0
	Intercept of SPLA curve	spla_intercept	(°C^-1^)	-250 – -100
	Curvature coefficient of specific plant leaf area	spla_prod_coef	(°C^-1^)	0.001–0.010
	Propensity to Tiller	tilleringPropensity	(-)	1.5–50
	Tiller Supply/demand slope	tillerSdSlope	(-)	0.1–0.5
	Inflection coefficient of TPLA curve	tpla_inflection_ratio	(°C)	0.5–0.9
	Curvature coefficient for leaf area	tpla_prod_coef	(°C^-1^)	0.01–0.04
	TT (thermal time) from emergence to end of juvenile	tt_emerg_to_endjuv	(°C d^-1^)	100–500
	TT from end of juvenile to floral initiation	tt_endjuv_to_init	(°C d^-1^)	50–500
	TT from flag leaf to flowering	tt_flag_to_flower	(°C d^-1^)	50–500
	TT from flowering to maturity	tt_flower_to_maturity	(°C d^-1^)	200–800
	TT from flowering to start grain fill	tt_flower_to_start_grain	(°C d^-1^)	30–500
	TT from maturity to ripe	tt_maturity_to_ripe	(°C d^-1^)	1–100
**Rice**	Development rate in the photoperiod-sensitive phase	DVRI	(°C d^-1^)	0.000750 - 0.001100
	Development rate in the juvenile phase	DVRJ	(°C d^-1^)	0.000773 - 0.001231
	Development rate in the panicle development phase	DVRP	(°C d^-1^)	0.000719 - 0.001600
	Development rate in the reproductive phase	DVRR	(°C d^-1^)	0.001244 - 0.002184
	Min. value of the relative growth rate of the leaf area	RGRLMN	(°C d^-1^)	0.003013 - 0.005494
	Max. value of the relative growth rate of the leaf area	RGRLMX	(°C d^-1^)	0.0060 - 0.0090
	Maximum individual grain weight	WGRMX	(mg grain^-1^)	19.0 - 30.0

For each simulation year output separate emulators were constructed in GEM-SA, resulting in 16 emulators for pearl millet (eight years each for yield and biomass) and 30 emulators for rice (eight years for HDS and seven years for WS, each for yield and biomass). Parameter influence was assessed through the parameter main effect (S_i_) and total effect (ST_i_) sensitivity indices. S_i_ and ST_i_ were calculated as follows.


$$S_{i}=\frac{Var\left\{E\left(f\left(X\;\;\;|\;\;\;x_{i}\right)\right)\right\}}{Var\left\{f\left(X\right)\right\}}$$ (2)

$$ST_{i}=1-\frac{Var\left\{E\left(f\left(X\;\;\;|\;\;\;x_{-i}\right)\right)\right\}}{Var\left\{f\left(X\right)\right\}}$$ (3)

where Var{f(X)} is the total variance in the output given variations in all parameters; $Var\left\{E\left(f\left(X\;\;\;|\;\;\;x_{i}\right)\right)\right\}$ is the variance in the expected output f(X) given $x_{i}$ is known; and $Var\left\{E\left(f\left(X\;\;\;|\;\;\;x_{-i}\right)\right)\right\}$ is the variance in the expected output f(X) if all parameters except $x_{i}$ are known.

### 2.3. Data analysis

#### 2.3.1. Model performance evaluation.

Model performance was evaluated through the calculation of the slope (α), intercept (β) and coefficient of determination (r^2^) of the linear regression between paired observed and predicted data-points. An intercept β of 0, and slope α and r^2^ of 1 indicate a perfect model fit. We also used the Student’s t-test of means assuming unequal variance to test for any statistical significance between observed and predicted values. p ≥ 0.05 indicates no statistically significant differences exist between the values. Wherever applicable, comparisons of means were performed using the Tukey’s HSD test at a 95% confidence level. Additional statistical model performance indicators used in this study included the absolute root mean square error (RMSE_a_, Mg ha^-1^, Eq. (4)), the normalized RMSE (RMSE_n_, %, Eq. (5)), the mean absolute error (MAE, Mg ha^-1^, Eq. (6)), and Willmott’s index (WI, -, Eq. (7)):


$$\begin{array}{c} RMSE_{a}\;\;\;\;\;\;\;=\;\;\;\;\;\;\;\sqrt{\underset{\text{i=1}}{\overset{\text{n}}{\sum }}\;{\text{(}{\text{O}}_{\text{i}}\text{ - }\;\;\;\;\;\;{\text{E}}_{\text{i}}\text{)}}^{\text{2}}/n} \end{array}$$ (4)

$$\begin{array}{c} RMSE_{n}=\;\;\;\;\;\;\;\sqrt{\underset{\text{i=1}}{\overset{\text{n}}{\sum }}\;{\text{(}{\text{O}}_{\text{i}}\text{ - }\;\;\;\;\;\;{\text{E}}_{\text{i}}\text{)}}^{\text{2}}/\text{n}}\text{* }\;\;\;\;\;\text{ 100}/\bar{\text{O}} \end{array}$$ (5)

$$\text{MAE }\;\;\;\;\;\text{ = }\;\;\;\;\;\;\frac{1}{n}\sum_{\text{i=1}}^{\text{n}}\left|{\text{E}}_{\text{i}}\text{-}{\text{O}}_{\text{i}}\right|$$ (6)

$$\begin{array}{c} \text{WI }\;\;\;\;\;\;=\;\;\;\;\;\;\text{ 1 }\;\;\;\;\;\text{ - }\;\;\;\;\;\;\left[\;\;\;\;\;\;\;\sum {\mathrm{\backslash nolimits}}_{\text{i=1}}^{\text{n}}{\left({\text{E}}_{\text{i}}\text{ - }\;\;\;\;\;\;{\text{O}}_{\text{i}}\right)}^{\text{2}}/\sum {\mathrm{\backslash nolimits}}_{\text{i=1}}^{\text{n}}{\left(\left|{\text{E}}_{\text{i}}\text{ - }\;\;\;\;\;\bar{\text{O}}\right|\;\;\;\;\;\;\text{ + }\;\;\;\;\;\;\left|{\text{O}}_{\text{i}}\text{ - }\;\;\;\;\;\bar{\text{O}}\right|\right)}^{\text{2}}\right] \end{array}$$ (7)

where E_i_ and O_i_ are the predicted and observed values, respectively; *n* is the number of data-pairs; and Ō is the mean of the observed values.

The RMSE_a_ is ideally similar to or smaller than the standard deviation (SD) of the observed values, while the RMSE_n_ is ideally similar to the coefficient of variation (CV) of the observed values. The MAE shows the absolute difference between the predicted and observed values; a MAE of 0 denotes a perfect model fit, whereas a positive or negative MAE indicates an over- or underestimation. The WI represents the ratio of the mean square error and the potential error, with values ranging between 0 and 1. The perfect score for a good model fit is 1.

#### 2.3.2. Assessment of yield stability.

We used the adjusted coefficient of variance (aCV, Eq. (8)) [51], the sustainable yield index (SYI, Eq. (9)) [52,53], and the Finlay-Wilkinson (F-W) coefficient [54] to examine the yield stability for different management options in this study. The aCV was introduced by Döring and Reckling [51] to account for the systematic dependence of the variance from the mean. The aCV for a given treatment (or management option) *i* was calculated as follows [51]:


$$\begin{array}{c} \text{aC}{\text{V}}_{\text{i}}\;\;\;\;\;\;\;=\;\;\;\;\;\;\text{ 1}/{\bar{\text{Y}}}_{\text{i}}\;\;\;\;\;\;\text{ * }\;\;\;\;\;\;{\left[\text{1}{\text{0}}^{\left(\text{2-b}\right){\text{m}}_{\text{i}}\text{+ }\;\;\;\;\;\;\left(\text{b-2}\right)\text{\textbackslash{}bar\{m}+}{\text{v}}_{\text{i}}\}\right]}^{\text{0.5}}\text{* }\;\;\;\;\;\text{ 100} \end{array}$$ (8)

where ${\bar{Y}}_{i}$ is the mean yield for a given treatment (or management option) *i*; *m*_*i*_ is the log_10_(${\bar{Y}}_{i}$); $\bar{m}$ is the average value of all *m*_*i*_; *v*_*i*_ is the log_10_ of the yield variance $\sigma_{i}^{2}$ for *i*; and b is the model coefficient of the linear regression $\begin{array}{c} \text{lo}{\text{g}}_{\text{10}}\left({\;\;\;\sigma \;\;\;}^{\text{2}}\right)\;\;\;\;\;\;\;=\;\;\;\;\;\;\text{ a }\;\;\;\;\;\text{ + }\;\;\;\;\;\text{ b }\;\;\;\;\;\text{ * }\;\;\;\;\;\text{ lo}{\text{g}}_{\text{10}}\left(\bar{\text{Y}}\right) \end{array}$. High aCV values are indicative of reduced yield stability.

The SYI was calculated as follows [52,53]:


$$\text{SY}{\text{I}}_{\text{i}}\;\;\;\;\;\;\;=\;\;\;\;\;\;\;\left({\bar{Y}}_{\;\;\;\;\;\;\text{ i}}\text{- }\delta_{\text{i}}\right)/{\text{Y}}_{\text{max}}$$ (9)

where SYI_i_ is the sustainable yield index for a given treatment (or management option) *i*; *δ*_*i*_ is the standard deviation of yield for *i*; and *Y*_*max*_ is the maximum yield in all years and treatments (or management options). SYI values range between 0–1, with values closer to 0 indicating unsustainable management due to a high standard deviation, and values closer to 1 indicating sustainable management due to a low standard deviation.

Paired treatment and environmental mean yields were regressed using the F-W regression, ŋ_ij_ = β_i_ + α_i_ * ω_j_, where ŋ_ij_ is the modelled yield response to the i^th^ treatment in the j^th^ environment; β_i_ and α_i_ are intercept and slope for the i^th^ treatment, and ω_j_ is the mean yield in the e^th^ environment [15,54]. The slope α_i_ of the F-W regression, referred to as the F-W coefficient, can be interpreted as a measure of sensitivity with small absolute values indicating stable responses over changing environments [15].

We further evaluated whether there was agreement between yield stability measures calculated based on the LTEs data and crop model outputs. For this analysis, Lin’s concordance correlation was used. All statistical analyses were performed in R (v. 4.3.1) [55] within the RStudio development environment [56].

## 3. Results

### 3.1. Crop phenology

**Fig 2:**
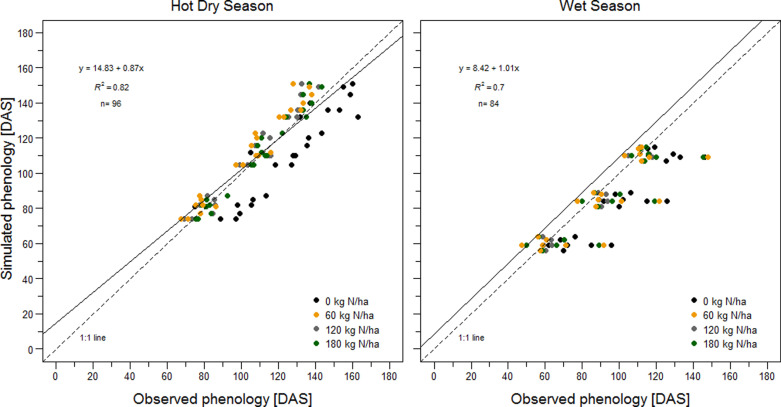
Comparison of the observed and simulated phenological stages of rice6 in Ndiaye, Senegal. (Left) Hot dry season and (right) wet season. Data for panicle initiation, flowering, and physiological maturity are presented. Encircled data-points show results from the 2020 season.

During the 2011–2018 experiments at Sadoré the average cropping season duration of pearl millet variety HKP was 97 ± 2 days. For APSIM-Millet simulations over the same period the cropping season lasted 96 ± 3 days on average (S3 Table). At Ndiaye, observed rice phenological development differed greatly among seasons, management options and years. During the HDS, observed days to flowering and physiological maturity ranged from 97 to 144 days after sowing (DAS), and from 120 to 163 DAS, respectively. The corresponding ranges during the WS were 78–126 DAS, and 103–148 DAS, respectively. In comparison, the average observed crop duration was 137 days during the HDS and 119 days during the WS. The differences in crop durations were reasonably well simulated, with an average of 140 days during the HDS and 111 days during the WS. Despite such marked seasonal differences in observed phenology, the overall model performance was acceptable, with a R^2^ of 0.82 during the HDS and of 0.70 during the WS (Fig 2). During model calibration, MAEs in days to maturity varied between 11 days during the HDS and 7 days during the WS, and between 7 days (HDS) and 12 days (WS) during model validation. However, the model failed to predict the extended crop duration of the 0 kg N ha^-1^ treatment during the HDS as compared to the other treatments (by between 16–21 days) (Fig 2). The model also missed the extended crop durations of all treatments by between 25–39 days during the 2020 WS season (Fig 2).

### 3.2. Biomass accumulation and grain yield

**Fig 3:**
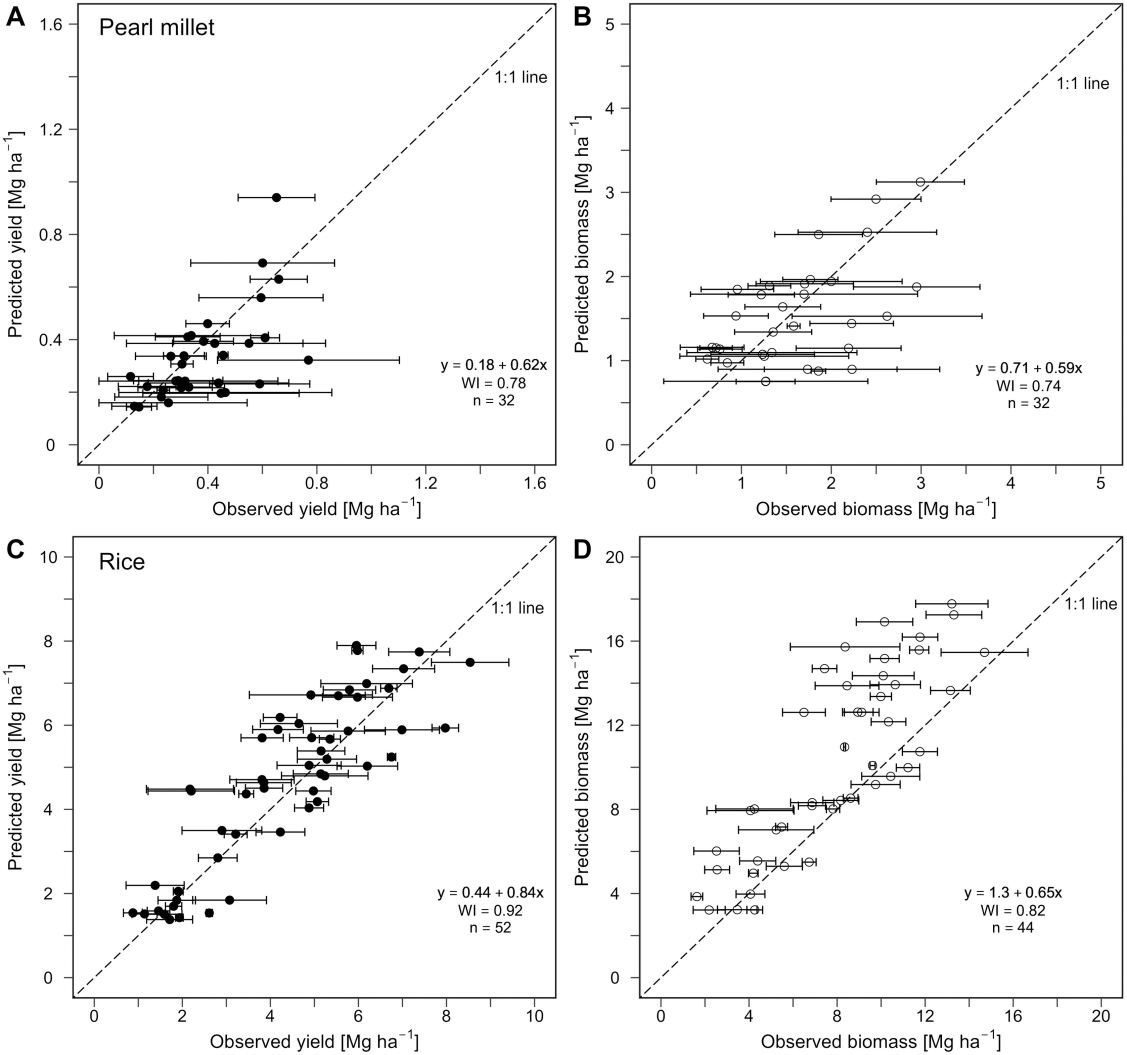
Performance evaluation of APSIM models during the validation step. Scatter plots of observed versus predicted pearl millet grain yield **(A)** and biomass **(B)**. Scatter plots of observed versus predicted rice grain yield **(C)** and biomass **(D)**. WI: Willmott’s index of agreement; n: number of data pairs. Residuals plots are presented in S2-S4 Figs for further visualization of error distribution.

The performance evaluations of APSIM models during the validation are presented in Table 6, Fig 3, and S1-S4 Figs. For APSIM-Millet simulations under CR_ret_, results revealed acceptable model error levels for grain yield, with WI = 0.74, MAE = 0.13 Mg ha^-1^, RMSE_a_ = 0.19 Mg ha^-1^ and RMSE_n_ of 46.0% (Fig 3A and Table 6). For biomass, the simulations were also in good agreement with the measurements; the values for WI, MAE, RMSE_a_ and RMSE_n_ were 0.77, 0.50 Mg ha^-1^ and 0.60 Mg ha^-1^, and 41.0%, respectively (Fig 3B and Table 6). The RMSE_a_ and RMSE_n_ for predicted yield and biomass were fairly similar to the SD and CV of observed values: for observed yields the SD in the validation dataset was 0.18 and the CV was 43.9%; for biomass they were 0.65 and 38.0%, respectively (Table 6). Moreover, the pairwise comparisons between the observed and predicted yield and biomass revealed no significant differences (p > 0.05), confirming acceptable model performance. When using data for plots under CR_rmv_ for APSIM-Millet second performance evaluation, the overall model performance was acceptable. For grain yield the WI was 0.62, and the MAE, RMSE_a_ and RMSE_n_ were 0.19 Mg ha^-1^, 0.24 Mg ha^-1^, and 59.0%, respectively; for biomass the corresponding performance statistics were 0.70, 0.65 Mg ha^-1^, 0.83 Mg ha^-1^, and 41.0%, respectively (Table 6; S1 Fig).

**Table 6:** Statistics for observed and predicted grain yield and aboveground biomass during the model calibration and validation steps for pearl millet and rice.

Crop	Model evaluation	Parameter	*n*	X_obs_ (SD) [Mg ha^-1^]	X_pred_ (SD) [Mg ha^-1^]	CV [%]	alpha α []	beta β [Mg ha^-1^]	R^2^ [0–1]	P(t)^*^	MAE [Mg ha^-1^]	RMSE_a_ [Mg ha^-1^]	RMSE_n_ [%]	WI [0–1]
**Pearl Millet**	calibration	grain	8	0.47 (0.16)	0.40 (0.20)	34.0	0.26	0.52	0.39	0.27	0.13	0.16	34.0	0.76
		biomass	8	1.98 (0.46)	1.76 (0.62)	23.2	1.30	0.39	0.28	0.30	0.47	0.55	28.0	0.69
	validation (CR retention)	grain	40	0.41 (0.18)	0.36 (0.21)	43.9	0.24	0.48	0.34	0.09	0.13	0.19	46.0	0.74
		biomass	40	1.71 (0.65)	1.66 (0.69)	38.0	0.78	0.56	0.36	0.58	0.50	0.60	35.0	0.77
	validation (CR removal)	grain	48	0.41 (0.22)	0.41 (0.23)	53.7	0.24	0.40	0.16	0.97	0.19	0.24	59.0	0.62
		biomass	48	2.01 (0.92)	1.83 (0.69)	45.8	0.78	0.67	0.26	0.13	0.65	0.83	41.0	0.70
**Rice**	calibration	grain	*8*	4.47 (1.28)	4.86 (2.05)	28.7	0.59	1.6	0.89	0.31	0.95	1.01	22.68	0.91
		biomass	*8*	8.62 (2.07)	10.97 (4.45)	24.0	0.35	4.78	0.57	0.09	2.91	3.96	45.9	0.64
	validation	grain	*52*	4.33 (1.92)	4.63 (1.98)	44.3	0.84	0.44	0.75	0.05	0.84	1.05	24.3	0.92
		biomass	*44*	7.91 (3.34)	10.14 (4.32)	42.2	0.65	1.34	0.64	0.00	2.53	3.25	41.1	0.82

*n*, number of data pairs; X_obs_ and X_pred_, mean of observed and predicted values; SD, standard deviation; CV, coefficient of variation; α and β, slope and intercept of linear regression; R^2^, coefficient of determination; P(t*), significance of Student’s paired *t*-test assuming non-equal variances; MAE, mean absolute error; RMSEa and RMSEn, absolute and normalized root mean square error; WI, Willmott’s index.

*values greater than 0.05 indicate simulated and observed values are the same at 95% confidence level.

For rice, a linear regression of observed and predicted data-pairs for grain yield illustrates acceptable model performance, with a strong correlation (R^2^ = 0.75), a mild bias (α = 0.84, β = 0.44 Mg ha^-1^), and a low MAE of 0.84 Mg ha^-1^ (Fig 3C). This model performance was further supported by the WI and RMSE statistics: a WI of 0.92 and RMSE_a_ of 1.05 Mg ha^-1^ and RMSE_n_ of 24.3%, which compared favorably with the SD of observed yields (1.92 Mg ha^-1^) and CV (44.3%), respectively (Table 6). Additionally, the paired Student’s *t*-test (assuming non-equal variances) confirmed that there is no statistical difference (p ≥ 0.05) between observed and predicted rice yields (Table 6).

### 3.3. Sensitivity of yield and biomass to crop parameters

**Fig 4:**
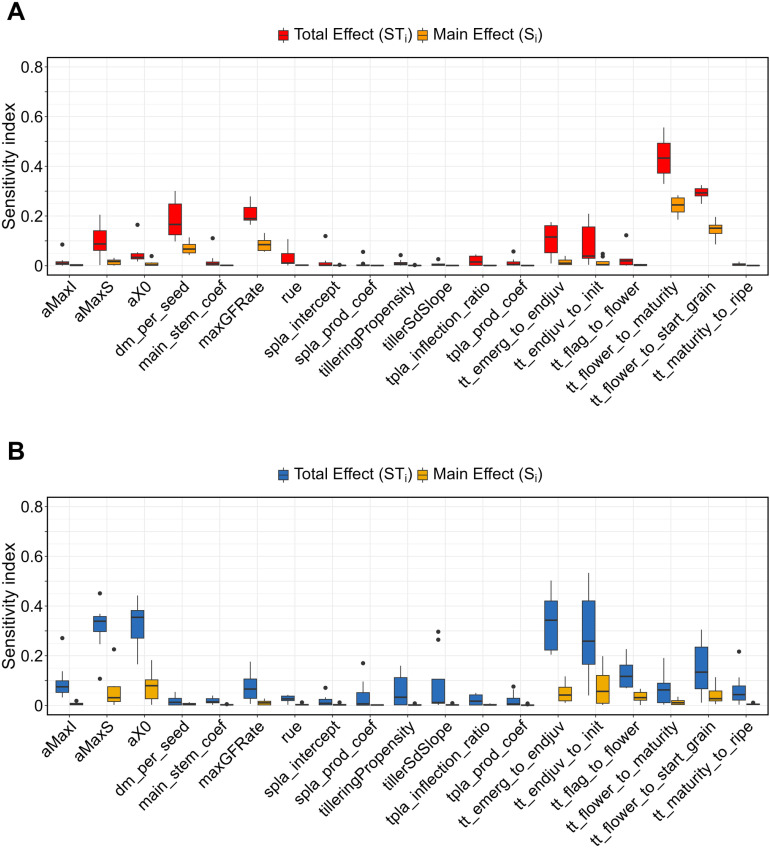
Distribution of the total effect (ST_i_) and main effect (S_i_) indices for 19 parameters across 8 years (2011-2018) for pearl millet in Sadoré, Niger. **(A)** Yield and **(B)** biomass outputs. In a boxplot, the top and bottom of the box represent the 75^th^ and 25^th^ percentiles, respectively. The solid line within the box indicates the median. The whiskers extend to the maximum and minimum excluding outliers. Outliers are shown as black circles.

Pearl millet yield was more sensitive to tt_flower_to_start_grain and tt_flower_to_maturity parameters, with both S_i_ and ST_i_ indices ≥ 0.1 (Fig 4A). In addition to these two parameters, for aMaxS, dm_per_seed, maxGFRate, tt_endjuv_to_init and tt_emerg_to_endjuv, parameters, ST_i_ index values differed notably from S_i_ index values (Fig 4A), indicating interactions between parameters. Similar patterns were found for pearl millet biomass for the parameters aMaxS, aX0, tt_endjuv_to_init, tt_emerg_to_endjuv, and tt_flower_to_start_grain parameters (Fig 4B). These parameters were also the most influential of pearl millet biomass, as indicated by their S_i_ index values (median S_i_ values above 0.05; Fig 4B).

**Fig 5:**
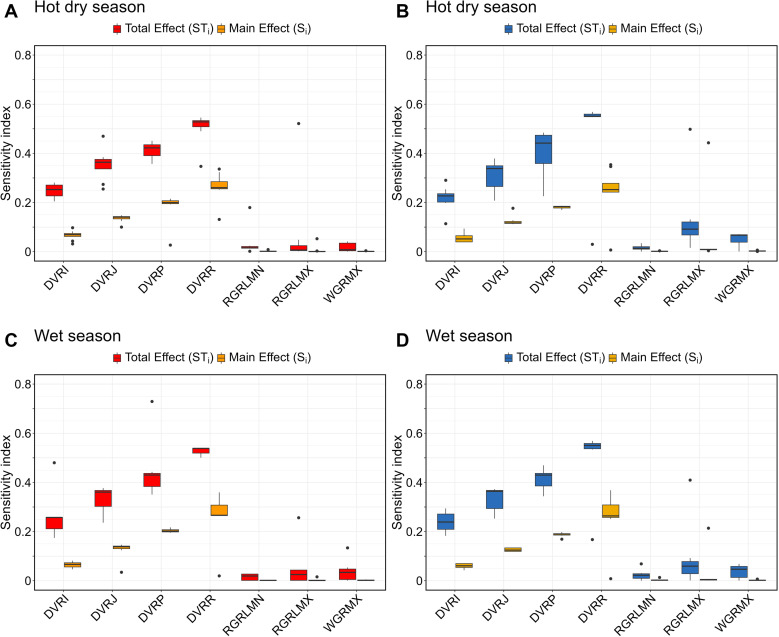
Distribution of the total effect (ST_i_) and main effect (S_i_) indices for 7 parameters across 8 years (2015-2022) for rice in Ndiaye, Senegal. Top: Hot dry season **(A)** yield and **(B)** biomass outputs. Bottom: Wet season **(C)** yield and **(D)** biomass outputs. In a boxplot, the top and bottom of the box represent the 75^th^ and 25^th^ percentiles, respectively. The solid line within the box indicates the median. The whiskers extend to the maximum and minimum excluding outliers. Outliers are shown as black circles.

For rice, similar patterns were found for the seven parameters evaluated in both HDS and WS (Fig 5). Both yield and biomass were more sensitive to DVRR, DVRP, DVRJ, and DVRI, with DVRR having the highest S_i_ index value. Similar to the two most influential parameters of pearl millet yield (tt_flower_to_start_grain and tt_flower_to_maturity), these five parameters interacted between them, as indicated by the marked difference between their ST_i_ and S_i_ values (Fig 5). Less influential parameters of rice biomass —RGRLMX and WGRMX— also exhibited notable differences between their ST_i_ and S_i_ index values in both cropping seasons (Fig 5B and 5D). This observation indicates that the sensitivity of APSIM-Oryza outputs to these parameters was primarily influenced by interactions with other parameters. Values of calibrated crop parameters used for pearl millet and rice simulations are provided in S1 and S2 Tables, respectively.

### 3.4. Yield variability and stability

The observed and predicted pearl millet yields from 2011 to 2018 showed that the control (T0) plots had the lowest grain yield, irrespective of the plant density and crop residue management (Table 7). Average observed yields in T1 and T2 plots under crop residue retention (CR_ret_) were higher, ranging from 0.46 ± 0.19 to 0.52 ± 0.18 Mg ha^-1^ (Table 7). Although average yields in T0 were the lowest, they did not statistically differ from those in fertilizer-treated plots (p > 0.5) when comparing within plant density groups (Table 7). The only statistical difference (p < 0.01) was found between T1/T2 plots in PDENS1 and T0 plot in PDENS2 (Table 7). In plots under crop residue removal (CR_rmv_) average observed yields varied between 0.26 ± 0.19 and 0.55 ± 0.22 Mg ha^-1^. Yields were statistically similar among treatments due to high variability, which is reflected through SD values. Only yields in T1 and T0 under PDENS1 were statistically different (p < 0.05; Table 7). As for the predicted yields, there was a greater variability (with SD up to 0.32 Mg ha^-1^) in T1 and T2 compared to T0 (SD = 0.10 Mg ha^-1^), with average yields ranging from 0.25 ± 0.10 to 0.48 ± 0.31 Mg ha^-1^ for CR_ret_, and from 0.28 ± 0.10 to 0.52 ± 0.32 Mg ha^-1^ for CR_rmv_ (Table 7).

**Table 7:** Yield stability measures of observed and predicted grain yields during model evaluation for pearl millet in Sadoré, Niger, and rice in Ndiaye, Senegal. Values are the means of the respective *n*. Treatments with the same letter are not significantly different (p > 0.05).

Crop	N rate [kg ha-1]	PDENS/ Season	CR	n Df	Yieldobs (SD) [Mg ha-1]		Yieldpred (SD) [Mg ha-1]		aCVobs [%]	aCVpred [%]	SYIobs [-]	SYIpred [-]
**Pearl Millet**	T0	PDENS1	CR_ret_	8	0.30 (0.11)	^ab^	0.25 (0.10)	^a^	31.6	51.9	0.25	0.13
	T1	PDENS1	CR_ret_	8	0.52 (0.13)	^a^	0.44 (0.24)	^a^	27.1	48.8	0.51	0.18
	T2	PDENS1	CR_ret_	8	0.52 (0.18)	^a^	0.48 (0.31)	^a^	37.3	53.9	0.43	0.15
	T0	PDENS2	CR_ret_	8	0.26 (0.10)	^b^	0.27 (0.11)	^a^	37.7	45.1	0.21	0.20
	T1	PDENS2	CR_ret_	8	0.46 (0.19)	^ab^	0.37 (0.17)	^a^	40.6	43.5	0.36	0.26
	T2	PDENS2	CR_ret_	8	0.47 (0.16)	^ab^	0.40 (0.20)	^a^	35.2	46.0	0.39	0.25
	T0	PDENS1	CR_rmv_	8	0.26 (0.19)	^b^	0.28 (0.10)	^a^	51.7	50.0	0.07	0.15
	T1	PDENS1	CR_rmv_	8	0.55 (0.22)	^a^	0.49 (0.28)	^a^	48.6	50.0	0.37	0.17
	T2	PDENS1	CR_rmv_	8	0.50 (0.25)	^ab^	0.52 (0.32)	^a^	56.6	50.1	0.28	0.16
	T0	PDENS2	CR_rmv_	8	0.28 (0.23)	^ab^	0.28 (0.09)	^a^	52.7	40.1	0.07	0.22
	T1	PDENS2	CR_rmv_	8	0.39 (0.16)	^ab^	0.42 (0.18)	^a^	43.6	39.8	0.35	0.28
	T2	PDENS2	CR_rmv_	8	0.46 (0.18)	^ab^	0.45 (0.20)	^a^	55.4	40.4	0.42	0.29
*ANOVA probabilities for the effects of* ^*a*^		CR_ret_	N rate (N) ×PDENS	5	4.458	**	1.760	ns				
			Year	1	3293	ns	10.572	**				
			N×Year	2	0.308	ns	0.0559	ns				
		CR_rmv_	N×PDENS	5	2.695	*	1.964	ns				
			Year	1	2.854	ns	5.730	ns				
			N×Year	2	2.200	ns	0.254	ns				
**Rice**	0	HDS		8	2.3 (0.8)	^c^	2.0 (0.4)	^c^	27.1	13.9	0.18	0.19
	60	HDS		8	5.1 (0.7)	^b^	5.2 (0.4)	^b^	14.9	8.1	0.51	0.61
	120	HDS		8	5.9 (1.0)	^ab^	6.5 (0.5)	^a^	21.4	10.2	0.57	0.76
	180	HDS		8	6.6 (1.1)	^a^	7.5 (0.4)	^a^	21.0	8.4	0.65	0.89
	0	WS		7	1.6 (0.5)	^b^	1.6 (0.2)	^b^	19.2	7.9	0.13	0.17
	60	WS		7	3.8 (0.9)	^a^	3.9 (0.4)	^a^	22.6	9.7	0.35	0.44
	120	WS		7	4.5 (1.4)	^a^	5.0 (0.5)	^a^	33.6	11.6	0.36	0.57
	180	WS		7	4.6 (1.5)	^a^	5.4 (0.8)	^a^	34.1	16.5	0.37	0.59
*ANOVA probabilities for the effects of*			N rate (N)	3	38.6	***	294.4	***				
			Season	1	25.2	***	116.1	***				
			Year	1	6.519	ns	15.185	ns				
			N×Season	3	1.1	ns	8.0	***				
			N×Year	3	0.287	ns	0.386	ns				
			Year×Season	1	0.031	ns	0.780	ns				

N rate: applied Urea N rate; Variable 1: plant density in pearl millet and season in rice; Variable 2: residue management option in pearl millet [CR retention (CR_ret_) and CR removal (CR_rmv_)]; PDENS1 and PDENS2, plant density 1 and plant density 2, respectively. *n*, number of data pairs; Yield_obs_ and Yield_pred_: mean of observed and predicted grain yields, respectively; SD: standard deviation; aCV_obs_ and aCV_pred_: adjusted coefficient of variance for observed and predicted grain yields, respectively; SYI_obs_ and SYI_pred_: sustainable yield index for observed and predicted grain yields, respectively.

^a^For pearl millet the ANOVA was performed according to the CR treatment for each yield dataset (observed and predicted) since there was no significant N×PDENS×CR interaction effect (p = 0.697 and 0.988, F-values = 0.363 and 0.012 in observed and predicted data, respectively).

Statistical significance codes: ns = not significant; *** denotes a p-value < 0.001, ** p-value < 0.01, * p-value < 0.5.

Pearl millet yield stability assessment using the aCV indicated that T2 had lower stability (aCV = 37.3%) compared to T0 (aCV = 31.6%) and T1 (aCV = 27.1%) in plots under PDENS1 and CR_ret_ between 2011 and 2018 (Table 7). However, under increased plant density (PDENS2) with CR_ret_, T2 was more stable (aCV = 35.2%). For plots under CR_rmv_, T1 was more stable in both PDENS1 and PDENS2, with aCV = 48.6% and 43.6%, respectively. From APSIM-Millet simulations, all three fertilizer treatments resulted in similar aCVs for PDENS1 under CR_rmv_. In PDENS2 under CR_rmv_ T1 had the lowest aCV (39.8%), but the difference from the highest aCV (40.4%), which was found for T2, was 0.6 percentage point (Table 7). Regarding CR_ret_, T1 resulted in more stable yields in both plant densities, whereas T2 was the fertilizer treatment with reduced yield stability (Table 7). Moreover, fertilizer treatments in PDENS2 were more stable (i.e., had lower aCV) than those in PDENS1.

Yield stability assessment using the SYI showed that T1 and T2 had higher SYI values compared to T0 across plant densities for both observations and model simulations, suggesting more stable yields. For the observed data, SYI values were higher in T1 than in T2 for plots under PDENS1 in both CR_ret_ and CR_rmv_: SYI = 0.51 and 0.37 for T1 in CR_ret_ and CR_rmv_, respectively; their corresponding values for T2 being 0.43 and 0.28, respectively (Table 7). Conversely, for plots under PDENS2 T2 resulted in more stable yields than T1 in both CR_ret_ and CR_rmv_. For the model-estimated data, the pattern was the same for PDENS1 (SYI in T1 slightly higher than that in T2), but different for PDENS2. Indeed, there was no difference between T1 and T2 in terms of yield stability according to the SYI in each of the CR management options as these values were similar (Table 7).

**Fig 6:**
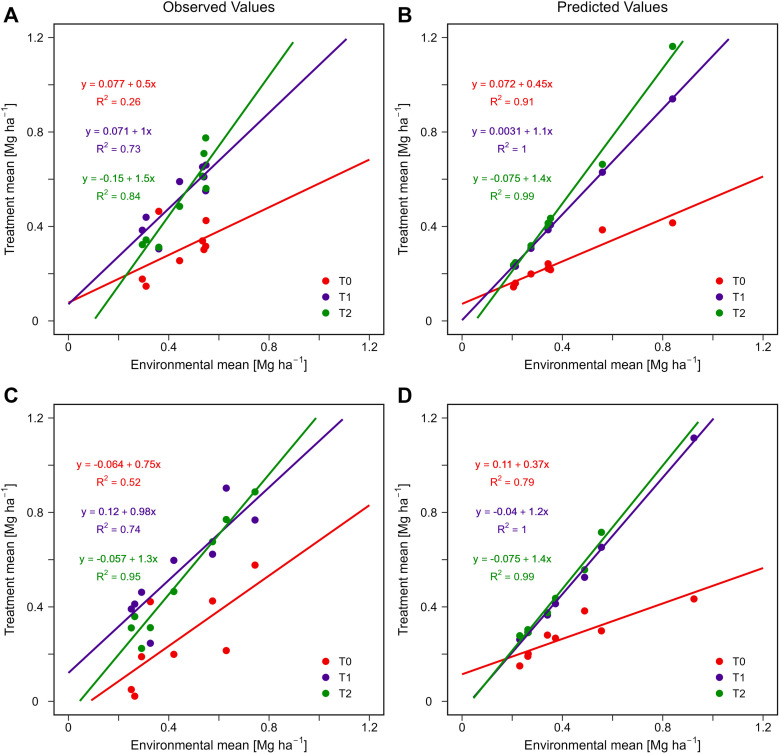
Finlay-Wilkinson regressions for pearl millet grain yield for different treatments. (Top) Yields of plots under crop residue retention: (**A**) observed and (**B**) predicted values. (Bottom) Yields of plots under crop residue removal: (**C**) observed and (**D**) predicted values. Data for plant density PDENS1 (10,000 pockets ha^-1^) are presented. T0 = control plot; T1 and T2: plots under fertilizer treatment T1 and T2, respectively.

Finlay-Wilkinson regression analysis perspective indicated that T0 in PDENS1 had the lowest slope coefficients across both crop residue management options, suggesting lower but more stable yields (slope coefficients ≤ 0.37) (Fig 6). In PDENS2 the fertilizer treated plots T1 and T2 resulted in higher-than-average yields, but reduced yield stability under CR_ret_ (slope coefficients > 1; S5 Fig). This pattern was observed in plots under CR_rmv_ for the predicted data and not the observed. For these latter values, T1 was the fertilizer treatment with a low F-W coefficient (slope coefficient = 0.82; S5 Fig).

**Fig 7:**
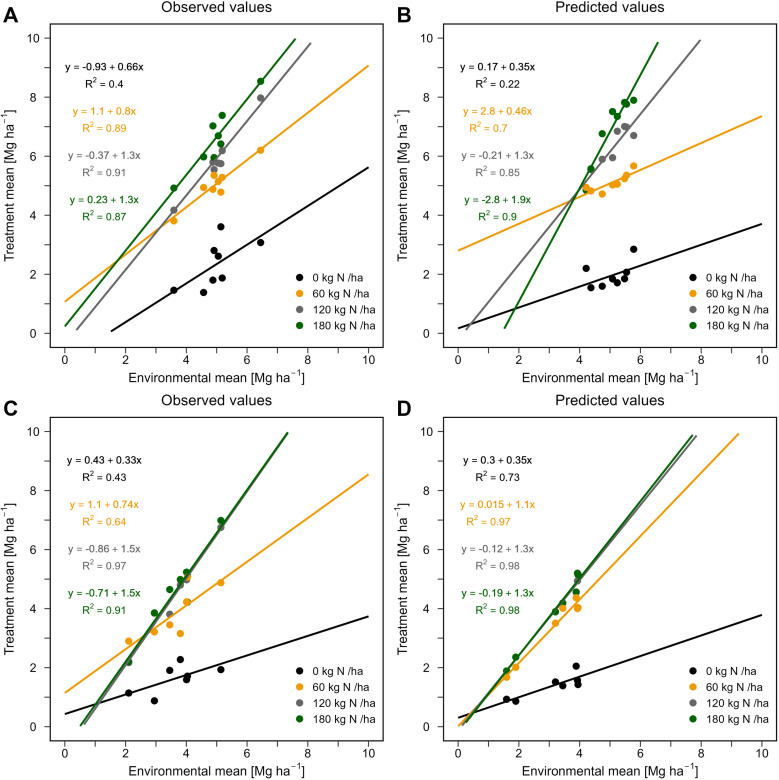
Finlay-Wilkinson regressions for rice grain yield for different treatments. (Top) Yields for the hot dry season: (**A**) observed and (**B**) predicted values. (Bottom) Yields for the wet season: (**C**) observed and (**D**) predicted values.

For rice, the observed and predicted mean yields in the HDS (5.0 and 5.3 Mg ha^-1^, respectively) were higher and statistically different (p < 0.001) than their correspondents in the WS (3.6 and 4.0 Mg ha^-1^, respectively). Irrespective of the cropping season, rice yield was more variable in the observed data, with SD ranging between 0.5 and 1.5 Mg ha^-1^, compared to predicted data (SD range of 0.4–0.8 Mg ha^-1^) (Table 7). In both the observed and predicted data, yield SD was comparatively higher in the WS than the HDS at N rates ≥ 120 kg N ha^-1^. Meanwhile, the aCV varied between 14.9 and 34.1% in the observed, and between 7.9 and 16.5% in the predicted data (Table 7). While the aCV increased proportionally with N rate during the WS, from 19.2 to 34.1% for observed yields, and from 7.9 to 16.5% for predicted yields, its pattern differed with increasing N rate during the HDS (Table 7). Indeed, for both observed and predicted yields, its values showed a non-linear trend with fluctuations: a decrease from 0 to 60 kg N ha^-1^, followed by a slight increase from 60 to 120 kg N ha^-1^, then a slight decrease from 120 to 180 kg N ha^-1^ (Table 7). Unlike the aCV, the SYI for observed and predicted yields increased proportionally with N rates in both the HDS and WS. The SYI indicated higher rice yield stability during the HDS, with values ranging from 0.18 to 0.65 and 0.19 to 0.89 for observed and predicted yields, respectively. Comparatively, during the WS, the SYI ranges for observed and predicted yields were 0.13–0.37 and 0.17–0.59, respectively (Table 7). The F-W regression indicates more stable yield responses to environmental changes in the low N treatments (0–60 kg N ha^-1^) with a slope coefficient α < 1 (between 0.49 and 0.82 in the observed, and between 0.36 and 0.99 in the predicted data) (Fig 7). Meanwhile, the observed and predicted yields of the 120 and 180 kg N ha^-1^ treatments had higher-than-average mean yields (slope coefficients > 1; Fig 7) but exhibited a lower yield stability according to the F-W stability measure.

### 3.5. Comparisons of yield stability measures for LTEs and model-estimated yields

Agreements between yield stability measures based on LTEs and model-estimated data are presented in Table 8. For pearl millet the F-W coefficient showed higher LCC and Cb values (0.70 and 0.97, respectively) compared to SYI and aCV when data were pooled. When analyzing treatment wise, good agreements were found for F-W coefficient in most cases (3 out of 4 treatments), with LCC coefficients ≥ 0.73 and Cb ≥ 0.80 (Table 8). For aCV there was no agreement between the stability calculated from the LTE and APSIM-Millet outputs; LCC coefficient and Cb values were close to 0 in all cases (Table 8) for aCV. This indicates a challenge in capturing yield stability through this measure for pearl millet during the study period. Similarly in rice, the aCV measure did not indicate good agreement in all cases, with LCC coefficient and Cb values being the lowest (≤ 0.16). However, both SYI and F-W coefficient demonstrated high agreement between yield stability calculated from the LTE and APSIM-Oryza outputs; LCC coefficients and Cb ranged from 0.56 and 0.78 and from 0.58 and 0.97, respectively (Table 8).

**Table 8:** Comparisons of yield stability measures for LTEs and model-estimated yields in pearl millet and rice across treatments and seasons.

Crop	Treatment/Season^a^	Yield stability measure	N	r	LCC coefficient	Cb^b^
**Pearl millet**	All	aCV	12	0.32	-0.23	0.72
	All	SYI	12	0.24	0.11	0.44
	All	F-W coef.	12	0.72	0.70	0.97
	PDENS1 | CR_ret_	aCV	3	0.98	0.04	0.04
	PDENS1 | CR_ret_	SYI	3	0.94	0.06	0.06
	PDENS1 | CR_ret_	F-W coef.	3	0.98	0.98	1.00
	PDENS2 | CR_ret_	aCV	3	0.99	-0.08	0.08
	PDENS2 | CR_ret_	SYI	3	0.95	0.28	0.30
	PDENS2 | CR_ret_	F-W coef.	3	0.89	0.86	0.97
	PDENS1 | CR_rmv_	aCV	3	0.92	0.02	0.02
	PDENS1 | CR_rmv_	SYI	3	0.97	0.09	0.09
	PDENS1 | CR_rmv_	F-W coef.	3	0.91	0.73	0.80
	PDENS2 | CR_rmv_	aCV	3	0.95	0.02	0.02
	PDENS2 | CR_rmv_	SYI	3	1.00	0.39	0.39
	PDENS2 | CR_rmv_	F-W coef.	3	0.57	-0.38	0.66
**Rice**	All	aCV	8	0.84	0.13	0.16
	All	SYI	8	0.97	0.75	0.77
	All	F-W coef.	8	0.81	0.78	0.97
	HDS	aCV	4	0.89	0.12	0.14
	HDS	SYI	4	0.99	0.78	0.79
	HDS	F-W coef.	4	0.94	0.71	0.75
	WS	aCV	4	0.85	0.12	0.14
	WS	SYI	4	0.96	0.56	0.58
	WS	F-W coef.	4	0.90	0.87	0.97

Abbreviations. N: number of data points. r = correlation coefficient; aCV: adjusted coefficient of variation. SYI: sustainability yield index. F-W coef.: Finlay-Wilkinson coefficient. LCC: Lin’s concordance correlation.

^a^PDENS1 and PDENS2 refer to plant density 1 and 2, respectively. CR_ret_: crop residue retention; CR_rmv_: crop residue removal. HDS: hot dry season; WS: wet season.

^b^Cb: LCC bias correction factor (1 = no bias).

## 4. Discussion

### 4.1. Contributions and comparisons with literature

We evaluated the performance of APSIM-Millet and APSIM-Oryza models in predicting grain yield and biomass of pearl millet and rice for locally recommended varieties in Niger and Senegal using LTEs data. We then evaluated the stability of various agronomic intensification options using the location-specific validated models. By calibrating and validating APSIM-Millet and APSIM-Oryza models with LTE data, we demonstrated the models’ capacity not only to predict average yields but also to replicate interannual yield variability. As such the study contributes to filling gaps in the modeling of rice and pearl millet systems under climate variability in the Sahel. Additionally, a direct comparison between yield stability metrics derived from field data and those from model outputs using concordance statistics (e.g., Lin’s concordance coefficient; Table 8) was performed. Through this comparison, we investigated how crop models reproduce observed yield stability patterns in pearl millet and rice. This approach assisted in evaluating the effectiveness of process-based models in resilience-oriented agronomy.

In terms of model performance, both models performed well within the experimental uncertainty at both study sites, which is a key measure of acceptable model performance [47]. APSIM-Millet simulated pearl millet yield across different residue management, fertilizer treatments and planting densities at the study site with acceptable error levels. This performance is comparable to that reported in [29] and [57]. In the former study, APSIM-Millet was used to assess the responses of pearl millet to different combined applications of cattle manure, crop residues and mineral fertilizers during two cropping seasons (1994–1995) at the same site in Niger. For grain yield the RMSE was 0.21 Mg ha^-1^ and the WI varied between 0.73 and 0.84; for the biomass the RMSE and WI ranged from 0.61 Mg ha^-1^ to 1.29 Mg ha^-1^, and 0.67 to 0.84, respectively [29]. In [57] the SARRAH (*Système d’Analyse Régionale des Risques Agroclimatiques, version H*) was tested for different pearl millet varieties, including HKP, in Burkina Faso, Mali, Niger, and Senegal, with the calibrated model explaining on average 34% of yield variability. Similar levels of model errors were also obtained with the Decision Support System for Agro-technological Transfer (DSSAT) CERES-Millet model when used under field conditions in different SSA countries [58,59].

### 4.2. Yield stability measures

In this study three measures including the SYI, the aCV, and the F-W coefficient were used to assess the stability of pearl millet and rice yields. We acknowledge that there is no clear consensus on the choice of yield stability measures as this is dependent of the research question and the understanding of the general properties of the stability measure [16]. Yield stability measures such as the CV and the standard error of regression model residuals were discarded since they can introduce potential bias in data interpretation, namely the well-known ‘mean-variance relationship’ in which treatments with low CV are not necessarily the more stable [51]. When assessing the ability of APSIM-Millet and APSIM-Oryza in capturing the trend in pearl millet and rice yield stability, the analysis showed that the patterns of SYI and F-W coefficient in model-estimated data were similar to those in LTEs data in most cases for both crops (Table 6). These two measures offered better agreement between the stability calculated from the LTEs and APSIM models outputs for pearl millet and rice (Table 8) and could henceforth be recommended for yield stability assessments. However, modeling studies aiming to design tailored management practices for higher and more stable yields, the use of the F-W coefficient needs to be carefully considered as its interpretation can be misleading. In this study, based on the F-W coefficient alone, control plots in both CR retention and CR removal in pearl millet were the most stable, though they had the lowest yields. Higher and more stable yields were obtained for treated plots under CR retention when assessing yield stability based on the SYI and aCV. To avoid misinterpretation, a set of at least two yield stability measures should be considered in crop modeling applications.

### 4.3. Model limitations

Model performance within the experimental uncertainty suggests that environment × management interactions and their effects on yield were simulated adequately at both study sites. However, some limitations in this study remain, indicating a need for further research. Our analysis showed that APSIM-Millet yield did not vary for P rates > 20 kg ha^-1^ (model version 7.10 r4220); only marginal changes were observed when comparing the 20 kg P ha^-1^ to 0 kg P ha^-1^. Without a sound calibration of soil P dynamics, the ability of the model to predict the impacts of P fertilizer on pearl millet growth and yield cannot be fully explored [60]. Besides, it has been shown that phosphorus positively affects pearl millet standing and yield under Sahelian conditions [61,62]. As such, the need for research on the modeling of P dynamics in APSIM-Millet is critically important to lend further confidence in the model ability to predict crop response to P-based fertilizers across West Africa. The same applies to modeling of K dynamics and its impact on yield.

Another limitation in pearl millet growth modeling is related to the lack of information on key phenological stages, e.g., flowering dates. While this might be related to the original objective of the experiments, i.e., pearl millet response to fertilizer microdose and crop residues management [4], this data unavailability prevented us from fully appreciating the model performance in predicting millet phenology at the study location. Although phenological data were collected in rice LTEs, we advocate for the monitoring of key phenological stages in field experiments to be fully integrated in the designs of LTEs to allow for better use of these data in modeling exercises. Such monitoring can be achieved through the integration of remote sensing technologies, such as high-resolution unmanned aerial vehicle imagery and satellite-derived phenology data, which can provide continuous and spatially explicit information on crop development stages. Moreover, the use of advanced crop and soil sensors for real-time monitoring of water and nutrient dynamics could improve the precision of model inputs and outputs.

Outputs from APSIM-Oryza for rice in Ndiaye showed discrepancies in predicting crop duration under specific conditions, particularly under low nitrogen input during the HDS and across all treatments in the 2020 WS season (Fig 2). These anomalies affected model accuracy; however, model performance improved markedly in their absence, with MAEs of 8 and 7 days to maturity during the HDS and WS, respectively. Integrating genomic data with process-based crop models offers a promising advancement for deepening our understanding of genotype-environment interactions [63]; this can help improve the performance of models in predicting rice phenology. Other means of improvement of performance include the integration of remote sensing data and machine learning methods with crop models [64,65].

### 4.4. Implications for future research

The sensitivity analysis revealed that pearl millet and rice yields and biomasses were most sensitive to phenological parameters, with sensitivity indices S_i_ and ST_i_ revealing strong individual and interactive effects (Figs 4 and 5). For both crops, notable differences between S_i_ and ST_i_ values across several parameters highlighted the importance of parameter interactions in driving model output variability. These findings underscore the need to consider both main effects and interactions in model calibration, particularly for parameters related to flowering and grain development stages in both crops. Moreover, to simplify the calibration process, parameters with low influence (low S_i_ and ST_i_ values), such as main_stem_coef, spla_intercept, spla_prod_coef, tpla_prod_coef, and tpla_inflection_ratio in pearl millet can be fixed at default values. APSIM models runs in the sensitivity analysis spanned eight years at both sites (2011–2018 for Sadoré, Niger, and 2015–2022 for Ndiaye, Senegal). Observed variations in index scores highlight the need to further investigate how climatic conditions may be contributing to the variability in sensitivity index. This aspect was not explored in this study and could be addressed in future research.

Our analysis showed that for rice that implementing season-specific nitrogen management strategies could reduce yield variability and enhance stability. For pearl millet fertilizer + CR retention increased yield stability, which is in line with the literature [66], highlighting the benefit of combining residue management with targeted fertilizer applications to mitigate inter-annual yield fluctuations. Investigating pearl millet response to different residue types was beyond the scope of this study. Exploring alternative residue type such as sorghum (*Sorghum bicolor* (L.) Moench), which is one of the major crops in Niger [2], or *Acacia tumida* or leguminous cover crops [67] could further improve soil properties and pearl millet yield stability. Indeed, sorghum and/or *A. tumida* residues can be used for mulching to reduce soil water evaporation, maintain soil moisture, and protect against erosion. Through their N-fixing capabilities, *A. tumida* and leguminous cover crops can enhance soil N supply, which could benefit pearl millet growth and grain yield.

## 5. Conclusions

We demonstrated the effectiveness of APSIM-Millet and APSIM-Oryza models in predicting grain yield and biomass of pearl millet and rice for locally-recommended varieties and various management options in Niger and Senegal. When assessing the ability of both models to capture yield stability trends in pearl millet and rice, our analysis showed that the SYI and Finlay-Wilkinson coefficient offered a better agreement between the stability calculated from the LTEs and APSIM models outputs, suggesting their suitability for yield stability assessments. The combined use of LTEs and APSIM models offers a comprehensive approach to understanding yield stability in pearl millet and rice systems. Well-tested crop models can help identify agronomic intensification options aimed at improving temporal yield stability in pearl millet and rice cropping systems in West Africa and elsewhere. These modeling approaches can be expanded to other LTEs across different regions and cropping systems, providing broader insights into agronomic interventions and their impact on yield stability. Future research could include yield stability assessments of management options under different climate change scenarios, which can serve as a basis for prioritizing research and development efforts.

## Supporting information

S1 Table Values of crop parameters used for the simulation of pearl millet cv. Haïni Kirey Précoce in APSIM-Millet. Parameters in bold were not calibrated, i.e., their values were those of cv. HHB 67–2 in the pearl millet module [35].
(PDF)

S2 Table APSIM phenological development parameters and biomass partitioning coefficients of the rice variety Sahel 108 in APSIM-Oryza. (PDF)

S3 Table Sowing and harvest dates of pearl millet during the study period 2011–2018. The predicted flowering and harvest dates using the calibrated APSIM-Millet are presented. Values in parentheses correspond to the days after sowing (DAS).
(PDF)

S1 Fig APSIM-Millet performance evaluation for plots under crop residue removal. (A) Scatterplots of observed versus predicted grain yield. (B) Scatterplots of observed versus predicted biomass. WI: Willmott’s index of agreement; n: number of data pairs. T0 = control plot; T1 and T2: plots under fertilizer treatment T1 and T2, respectively.
(TIF)

S2 Fig Residual plots for linear regression model between observed and predicted yield. Scatterplots of (A) fitted values vs residuals and (B) theoretical quantiles vs standardized residuals for pearl millet. Scatterplots of (C) fitted values vs residuals and (D) theoretical quantiles vs standardized residuals for rice. Pearl millet data are from plots under crop residue retention.
(TIF)

S3 Fig Residual plots for linear regression model between observed and predicted biomass. Scatterplots of (A) fitted values vs residuals and (B) theoretical quantiles vs standardized residuals for pearl millet. Scatterplots of (C) fitted values vs residuals and (D) theoretical quantiles vs standardized residuals for rice. Pearl millet data are from plots under crop residue retention.
(TIF)

S4 Fig Residual plots for linear regression model between observed and predicted pearl millet yield and biomass under crop residue removal. Scatterplots of (A) fitted values vs residuals and (B) theoretical quantiles vs standardized residuals for yield. Scatterplots of (C) fitted values vs residuals and (D) theoretical quantiles vs standardized residuals for biomass.
(TIF)

S5 Fig Finlay-Wilkinson regressions for pearl millet grain yield for different treatments. (Top) Plots under crop residue retention: (A) observed and (B) predicted yields. (Bottom) Plots under crop residue removal: (C) observed and (D) predicted yields. Data for plant density PDENS2 (15,000 pockets ha^-1^) are presented. T0 = control plot; T1 and T2: plots under fertilizer treatment T1 and T2, respectively.
(TIF)

## References

[1] Niang A, Becker M, Ewert F, Dieng I, Gaiser T, Tanaka A, et al. Variability and determinants of yields in rice production systems of West Africa. Field Crops Res. 2017;207:1–12. doi: 10.1016/j.fcr.2017.02.014

[2] Crops and livestock products [Internet]. Food and Agriculture Organization of the United Nations (FAO). 2024 [cited 2024 Jun 12]. Available from: https://www.fao.org/faostat/en/#data/QCL.

[3] de Rouw A. Improving yields and reducing risks in pearl millet farming in the African Sahel. Agric Syst. 2004;81(1):73–93. doi: 10.1016/j.agsy.2003.09.002

[4] Iliasso ADKT, Ali I, Dougbedji F, Jean Baptiste ED, Vincent B. Pearl millet yields and yield stability under long‐term soil fertility management in the Sahel. Agron J. 2022;114(4):2573–83. doi: 10.1002/agj2.21129

[5] Balasubramanian V, Sie M, Hijmans R, Otsuka K. Increasing rice production in sub-Saharan Africa: challenges and opportunities. In: Sparks D, editor. Advances in Agronomy. Academic Press. 2007. p. 55–133.

[6] Duvallet M, Dumas P, Makowski D, Boé J, del Villar PM, Ben-Ari T. Rice yield stability compared to major food crops in West Africa. Environ Res Lett. 2021;16(12):124005. doi: 10.1088/1748-9326/ac343a

[7] Senthilkumar K, Rodenburg J, Dieng I, Vandamme E, Sillo FS, Johnson J, et al. Quantifying rice yield gaps and their causes in Eastern and Southern Africa. J Agron Crop Science. 2020;206(4):478–90. doi: 10.1111/jac.12417

[8] Yuan S, Saito K, van Oort PAJ, van Ittersum MK, Peng S, Grassini P. Intensifying rice production to reduce imports and land conversion in Africa. Nat Commun. 2024;15(1):835. doi: 10.1038/s41467-024-44950-8 38280881 PMC10821910

[9] Arouna A, Fatognon IA, Saito K, Futakuchi K. Moving toward rice self-sufficiency in sub-Saharan Africa by 2030: lessons learned from 10 years of the coalition for African Rice development. World Dev Perspec. 2021;21:100291. doi: 10.1016/j.wdp.2021.100291 PMC7988505 33791446

[10] Krupnik TJ, Shennan C, Rodenburg J. Yield, water productivity and nutrient balances under the System of Rice Intensification and Recommended Management Practices in the Sahel. Field Crops Res. 2012;130:155–67. doi: 10.1016/j.fcr.2012.02.003

[11] Ibrahim A, Saito K, Bado VB, Wopereis MCS. Thirty years of agronomy research for development in irrigated rice-based cropping systems in the West African Sahel: achievements and perspectives. Field Crops Res. 2021;266:108149. doi: 10.1016/j.fcr.2021.108149

[12] Sarr B. Present and future climate change in the semi‐arid region of West Africa: a crucial input for practical adaptation in agriculture. Atmos Sci Lett. 2012;13(2):108–12. doi: 10.1002/asl.368

[13] Struik PC, Kuyper TW. Sustainable intensification in agriculture: the richer shade of green. A review. Agron Sustain Dev. 2017;37(5). doi: 10.1007/s13593-017-0445-7

[14] van Oort PAJ, Zwart SJ. Impacts of climate change on rice production in Africa and causes of simulated yield changes. Glob Chang Biol. 2018;24(3):1029–45. doi: 10.1111/gcb.13967 29230904 PMC5836867

[15] Skovbjerg CK, Knudsen JN, Füchtbauer W, Stougaard J, Stoddard FL, Janss L, et al. Evaluation of yield, yield stability, and yield–protein relationship in 17 commercial faba bean cultivars. Legume Sci. 2020;2(3). doi: 10.1002/leg3.39

[16] Reckling M, Ahrends H, Chen T-W, Eugster W, Hadasch S, Knapp S, et al. Methods of yield stability analysis in long-term field experiments. A review. Agron Sustain Dev. 2021;41(2). doi: 10.1007/s13593-021-00681-4

[17] Knapp S, van der Heijden MGA. A global meta-analysis of yield stability in organic and conservation agriculture. Nat Comm. 2018;9(1):3632. doi: 10.1038/s41467-018-05956-1 30194344 PMC6128901

[18] Grotelüschen K, Gaydon DS, Senthilkumar K, Langensiepen M, Becker M. Model-based evaluation of rainfed lowland rice responses to N fertiliser in variable hydro-edaphic wetlands of East Africa. Field Crops Res. 2022;285:108602. doi: 10.1016/j.fcr.2022.108602

[19] Macholdt J, Piepho H-P, Honermeier B, Perryman S, Macdonald A, Poulton P. The effects of cropping sequence, fertilization and straw management on the yield stability of winter wheat (1986–2017) in the Broadbalk Wheat Experiment, Rothamsted, UK. J Agric Sci. 2020;158(1–2):65–79. doi: 10.1017/s0021859620000301

[20] Reckling M, Döring TF, Bergkvist G, Stoddard FL, Watson CA, Seddig S, et al. Grain legume yields are as stable as other spring crops in long-term experiments across northern Europe. Agron Sustain Dev. 2018;38(6):63. doi: 10.1007/s13593-018-0541-3 30873223 PMC6390932

[21] Lobell DB, Schlenker W, Costa-Roberts J. Climate trends and global crop production since 1980. Science. 2011;333(6042):616–20. doi: 10.1126/science.1204531 21551030

[22] Silva JV, Giller KE. Grand challenges for the 21st century: what crop models can and can’t (yet) do. J Agric Sci. 2020;158(10):794–805. doi: 10.1017/s0021859621000150

[23] Jones JW, Antle JM, Basso B, Boote KJ, Conant RT, Foster I, et al. Brief history of agricultural systems modeling. Agric Syst. 2017;155:240–54. doi: 10.1016/j.agsy.2016.05.014 28701816 PMC5485640

[24] Grotelüschen K, Gaydon DS, Langensiepen M, Ziegler S, Kwesiga J, Senthilkumar K, et al. Assessing the effects of management and hydro-edaphic conditions on rice in contrasting East African wetlands using experimental and modelling approaches. Agric Water Manage. 2021;258:107146. doi: 10.1016/j.agwat.2021.107146

[25] Haefele SM, Wopereis MCS, Wiechmann H. Long-term fertility experiments for irrigated rice in the West African Sahel: agronomic results. Field Crops Res. 2002;78(2–3):119–31. doi: 10.1016/s0378-4290(02)00117-x

[26] Haefele SM, Thomas CL, Saito K. Long-term fertility experiments for irrigated rice in the West African Sahel: Effect on macro- and micronutrient concentrations in plant and soil. Field Crops Res. 2022;275:108357. doi: 10.1016/j.fcr.2021.108357

[27] West LT, Wilding LP, Landeck JK, Calhoun FG. Soil survey of the ICRISAT sahelian center, Niger, West Africa. International Crops Research Institute for the Semi-Arid Tropics (ICRISAT).

[28] Akponikpè PBI. Millet response to water and soil fertility management in the Sahelian Niger: experiments and modeling. Belgium: Universite Catholique de Louvain, Belgium; 2008. Available from: http://hdl.handle.net/2078.1/19624.

[29] Akponikpè PBI, Gérard B, Michels K, Bielders C. Use of the APSIM model in long term simulation to support decision making regarding nitrogen management for pearl millet in the Sahel. Eur J Agron. 2010;32(2):144–54. doi: 10.1016/j.eja.2009.09.005

[30] IRI, Michigan State University, IFPRI HarvestChoice. Global high-resolution soil profile database for crop modeling applications. In: International Research Institute for Climate and Society (IRI), University MS, HarvestChoice IFPRII, editors. V2 ed: Harvard Dataverse; p. doi: 10.7910/DVN/1PEEY0

[31] Dalgliesh N, Hochman Z, Huth N, Holzworth D. Field Protocol to APSoil characterisations. Version 4. CSIRO, Australia.

[32] Ruane AC, Goldberg R, Chryssanthacopoulos J. Climate forcing datasets for agricultural modeling: merged products for gap-filling and historical climate series estimation. Agric For Meteorol. 2015;200:233–48. doi: 10.1016/j.agrformet.2014.09.016

[33] Senthilkumar K, Tesha BJ, Mghase J, Rodenburg J. Increasing paddy yields and improving farm management: results from participatory experiments with good agricultural practices (GAP) in Tanzania. Paddy Water Environ. 2018;16(4):749–66. doi: 10.1007/s10333-018-0666-7

[34] Holzworth DP, Huth NI, deVoil PG, Zurcher EJ, Herrmann NI, McLean G, et al. APSIM – Evolution towards a new generation of agricultural systems simulation. Environ Model Softw. 2014;62:327–50. doi: 10.1016/j.envsoft.2014.07.009

[35] Garin V, van Oosterom E, McLean G, Hammer G, Murugesan T, Kaliamoorthy S, et al. New algorithm for pearl millet modelling in APSIM allowing a mechanistic simulation of tillers. BioRxiv [Preprint]. 2023 bioRxiv 528159 [posted 2023 Feb 13; revised 2023 Dec 5; cited 2024 Feb 5]: [17 p.]. Available from: http://biorxiv.org/content/early/2023/02/13/2023.02.12.528159.abstract.

[36] Hammer GL, McLean G, Kholová J, van Oosterom E. Modelling the dynamics and phenotypic consequences of tiller outgrowth and cessation in sorghum. in silico Plants. 2023;5(2):diad019. doi: 10.1093/insilicoplants/diad019

[37] Alam MM, van Oosterom EJ, Cruickshank AW, Jordan DR, Hammer GL. Predicting tillering of diverse sorghum germplasm across environments. Crop Sci. 2017;57(1):78–87. doi: 10.2135/cropsci2016.04.0262

[38] Bouman BAM, Kropff MJ, Wopereis MCS, ten Berge HFM, van Laar HH. ORYZA2000: modeling lowland rice. Los Baños, Philippines and Wageningen, Netherlands: International Rice Research Institute (IRRI) and Wageningen University. 2001.

[39] Zhang X, Lee J-H, Abawi Y, Kim Y, McClymont D, Kim H-D. Testing the simulation capability of APSIM-ORYZA under different levels of nitrogen fertiliser and transplanting time regimes in Korea. Aust J Exp Agric. 2007;47(12):1446. doi: 10.1071/ea05363

[40] Gaydon DS, Probert ME, Buresh RJ, Meinke H, Suriadi A, Dobermann A, et al. Rice in cropping systems—Modelling transitions between flooded and non-flooded soil environments. Eur J Agron. 2012;39:9–24. doi: 10.1016/j.eja.2012.01.003

[41] Gaydon DS, Probert ME, Buresh RJ, Meinke H, Timsina J. Modelling the role of algae in rice crop nutrition and soil organic carbon maintenance. European Journal of Agronomy. 2012;39:35–43. doi: 10.1016/j.eja.2012.01.004

[42] Probert ME, Dimes JP, Keating BA, Dalal RC, Strong WM. APSIM’s water and nitrogen modules and simulation of the dynamics of water and nitrogen in fallow systems. Agric Syst. 1998;56(1):1–28. doi: 10.1016/s0308-521x(97)00028-0

[43] Omanya GO, Weltzien-Rattunde E, Sogodogo D, Sanogo M, Hanssens N, Guero Y, et al. Participatory varietal selection with improved pearl millet in West Africa. Ex Agric. 2007;43(1):5–19. doi: 10.1017/s0014479706004248

[44] Miguez F. apsimx: Inspect, Read, Edit and Run ‘APSIM’ “Next Generation” and ‘APSIM’ Classic. R package. version 2.3.1. 2022.

[45] Bouman BAM, van Laar HH. Description and evaluation of the rice growth model ORYZA2000 under nitrogen-limited conditions. Agric Syst. 2006;87(3):249–73. doi: 10.1016/j.agsy.2004.09.011

[46] van Oort PAJ, Zhang T, de Vries ME, Heinemann AB, Meinke H. Correlation between temperature and phenology prediction error in rice (Oryza sativa L.). Agric Forest Meteorol. 2011;151(12):1545–55. doi: 10.1016/j.agrformet.2011.06.012

[47] Gaydon DS, Balwinder S, Wang E, Poulton PL, Ahmad B, Ahmed F, et al. Evaluation of the APSIM model in cropping systems of Asia. Field Crops Res. 2017;204:52–75. doi: 10.1016/j.fcr.2016.12.015

[48] Julia C, Dingkuhn M. Predicting temperature induced sterility of rice spikelets requires simulation of crop-generated microclimate. Eur J Agron. 2013;49:50–60. doi: 10.1016/j.eja.2013.03.006

[49] Kennedy MC, Petropoulos GP. Chapter 17 -GEM-SA: the gaussian emulation machine for sensitivity analysis. In: Petropoulos G, Srivastava P, editors. Sensitivity analysis in earth observation modelling. Amsterdam: Elsevier. 2017. p. 341–61.

[50] O’Hagan T. The GEM Software. 2017. [updated 11 January 2017]. Available from: https://www.tonyohagan.co.uk/academic/GEM/.

[51] Döring TF, Reckling M. Detecting global trends of cereal yield stability by adjusting the coefficient of variation. Eur J Agron. 2018;99:30–6. doi: 10.1016/j.eja.2018.06.007

[52] Singh RP, Das SK, Bhaskarrao UM, Reddy MN. Sustainability Index under Different Management: Annual Report. Hyderabad, India: CRIDA, 1990.

[53] Han X, Hu C, Chen Y, Qiao Y, Liu D, Fan J, et al. Crop yield stability and sustainability in a rice-wheat cropping system based on 34-year field experiment. Eur J Agron. 2020;113:125965. doi: 10.1016/j.eja.2019.125965

[54] Finlay KW, Wilkinson GN. The analysis of adaptation in a plant-breeding programme. Aust J Agric Res. 1963;14(6):742. doi: 10.1071/ar9630742

[55] R Core Team. R: A language and environment for statistical computing. Vienna, Austria: R Foundation for Statistical Computing; 2023. Available from: http://www.R-project.org/.

[56] Posit Team. RStudio: Integrated development environment for R. Boston, MA, USA: Posit Software, PBC. 2023.

[57] Traoré SB, Alhassane A, Muller B, Kouressy M, Somé L, Sultan B, et al. Characterizing and modeling the diversity of cropping situations under climatic constraints in West Africa. Atmos Sci Lett. 2010;12(1):89–95. doi: 10.1002/asl.295

[58] Soler CMT, Maman N, Zhang X, Mason SC, Hoogenboom G. Determining optimum planting dates for pearl millet for two contrasting environments using a modelling approach. J Agric Sci. 2008;146(4):445–59. doi: 10.1017/s0021859607007617

[59] Ullah A, Ahmad I, Ahmad A, Khaliq T, Saeed U, Habib-ur-Rahman M, et al. Assessing climate change impacts on pearl millet under arid and semi-arid environments using CSM-CERES-Millet model. Environ Sci Pollut Res. 2019;26(7):6745–57. doi: 10.1007/s11356-018-3925-7 30632035

[60] Raymond N, Kopittke PM, Wang E, Lester D, Bell MJ. Does the APSIM model capture soil phosphorus dynamics? A case study with Vertisols. Field Crops Res. 2021;273:108302. doi: 10.1016/j.fcr.2021.108302

[61] Bationo A, Payne WA, Renard C, Subbarao GV. Long-term effects of tillage, phosphorus fertilization and crop rotation on pearl millet–cowpea productivity in the West-African Sahel. Ex Agric. 2000;36(2):243–64. doi: 10.1017/s0014479700002106

[62] Bielders CL, Gérard B. Millet response to microdose fertilization in south–western Niger: effect of antecedent fertility management and environmental factors. Field Crops Res. 2015;171:165–75. doi: 10.1016/j.fcr.2014.10.008

[63] Yang Y, Wilson LT, Li T, Paleari L, Confalonieri R, Zhu Y, et al. Integration of genomics with crop modeling for predicting rice days to flowering: a multi-model analysis. Field Crops Res. 2022;276:108394. doi: 10.1016/j.fcr.2021.108394

[64] Zhang J, Lin X, Jiang C, Hu X, Liu B, Liu L, et al. Predicting rice phenology across China by integrating crop phenology model and machine learning. Sci Total Environ. 2024;951:175585. doi: 10.1016/j.scitotenv.2024.175585 39155002

[65] Brinkhoff J, McGavin SL, Dunn T, Dunn BW. Predicting rice phenology and optimal sowing dates in temperate regions using machine learning. Agron J. 2023;116(3):871–85. doi: 10.1002/agj2.21398

[66] Yamoah CF, Bationo A, Shapiro B, Koala S. Trend and stability analyses of millet yields treated with fertilizer and crop residues in the Sahel. Field Crops Res. 2002;75(1):53–62. doi: 10.1016/s0378-4290(02)00008-4

[67] Ibrahim A, Abaidoo RC, Fatondji D, Opoku A. Integrated use of fertilizer micro-dosing and *Acacia tumida* mulching increases millet yield and water use efficiency in Sahelian semi-arid environment. Nutr Cycl Agroecosyst. 2015;103(3):375–88. doi: 10.1007/s10705-015-9752-z

