# Supplementary material for: Assessing yield stability of pearl millet and rice cropping systems across West Africa using long-term experiments and a modeling approach
Source: PLoS One. 2025 May 27;20(5):e0317170. doi: 10.1371/journal.pone.0317170 (PMC12112412; doi:10.1371/journal.pone.0317170)
Supplement: S1 Table — Haïni Kirey Précoce in APSIM-Millet. Parameters in bold were not calibrated, i.e., their values were those of cv. HHB 67–2 in the pearl millet module [35]. (PDF) [file pone.0317170.s001.pdf]

S1 Table. Values of crop parameters used for the growth simulation of the pearl millet cultivar Haïni Kirey Précoce (HKP) in APSIM-Millet. Parameters in bold were not calibrated, i.e., their values were those of the cultivar HHB 67-2 in the pearl millet module [35].

**Parameter**	**Acronym**	**Unit**	**Pearl millet cv. HKP**
Intercept for largest leaf calculation	aMaxI	(-)	40.880120
Largest leaf Area Factor	aMaxS	(-)	6.50
Largest leaf multiplier	aX0	(-)	0.857056
Dry matter content per seed	dm_per_seed	(g)	0.000821
**Power coefficient for TPLAmax=TLN^coef**	**main_stem_coef**	(°C^{-1})	**2.79**
Maximum grain filling rate	maxGFRate	(mg °C d^{-1})	0.055202
**Intercept of SPLA curve**	**spla_intercept**	(°C^{-1})	**-170.92**
**Curvature coefficient of specific plant leaf area**	**spla_prod_coef**	(°C^{-1})	**0.008-**
**Tiller Supply/demand slope**	**tillerSdSlope**	(-)	**0.35**
**Propensity to Tiller**	**tilleringPropensity**	(-)	**3.4**
**Inflection coefficient of TPLA curve**	**tpla_inflection_ratio**	(°C)	**0.895**
**Curvature coefficient for leaf area**	**tpla_prod_coef**	(°C^{-1})	**0.02**
TT (thermal time) from emergence to end of juvenile	tt_emerg_to_endjuv	(°C d^{-1})	500
TT from end of juvenile to floral initiation	tt_endjuv_to_init	(°C d^{-1})	157
TT from flag leaf to flowering	tt_flag_to_flower	(°C d^{-1})	120
TT from flowering to maturity	tt_flower_to_maturity	(°C d^{-1})	430
TT from flowering to start grain fill	tt_flower_to_start_grain	(°C d^{-1})	81
**TT from maturity to ripe**	**tt_maturity_to_ripe**	**(°C d^{-1})**	**1**
