# Supplementary material for: Assessing yield stability of pearl millet and rice cropping systems across West Africa using long-term experiments and a modeling approach
Source: PLoS One. 2025 May 27;20(5):e0317170. doi: 10.1371/journal.pone.0317170 (PMC12112412; doi:10.1371/journal.pone.0317170)
Supplement: S2 Table — (PDF) [file pone.0317170.s002.pdf]

S2 Table. APSIM phenological development parameters and biomass partitioning coefficients of the rice variety Sahel 108 in APSIM-Oryza.

Parameter	Acronym	Unit	lowland rice cv. Sahel 108
Development rate in the photoperiod-sensitive phase	DVRI	(°C d^{-1})	0.000907
Development rate in the juvenile phase	DVRJ	(°C d^{-1})	0.000907
Development rate in the panicle development phase	DVRP	(°C d^{-1})	0.000985
Development rate in the reproductive phase	DVRR	(°C d^{-1})	0.002795
Min. value of the relative growth rate of the leaf area	RGRLMN	(°C d^{-1})	0.0040
Max. value of the relative growth rate of the leaf area	RGRLMX	(°C d^{-1})	0.0075
Maximum individual grain weight	WGRMX	(mg $grain^{-1}$)	23.0
Development stage	DVS	(0-2)	[0.00; 0.71; 1.17; 2.00; 2.50]
Fraction of shoot dry matter to leaves	FLV	(0-1)	[0.60; 0.22; 0.08; 0.00; 0.00]
Fraction of shoot dry matter to stems	FST	(0-1)	[0.40; 0.56; 0.08; 0.00; 0.00]
Fraction of shoot dry matter to storage organs	FSO	(0-1)	[0.00; 0.22; 0.90; 1.00; 1.00]
Leaf death coefficient	DRLV	(0-1)	[0.000; 0.001; 0.024; 0.030; 0.030]
	DVS	(0-2)	[0.00; 0.57; 0.85; 1.48; 1.95; 2.50]
Specific green leaf area	SLA	(m^2 leaf kg $leaf^{-1}$)	[0.0036; 0.0025; 0.0022; 0.0018; 0.0016; 0.0016]
