# Supplementary material for: Assessing yield stability of pearl millet and rice cropping systems across West Africa using long-term experiments and a modeling approach
Source: PLoS One. 2025 May 27;20(5):e0317170. doi: 10.1371/journal.pone.0317170 (PMC12112412; doi:10.1371/journal.pone.0317170)
Supplement: S3 Table — The predicted flowering and harvest dates using the calibrated APSIM-Millet are presented. Values in parentheses correspond to the days after sowing (DAS). (PDF) [file pone.0317170.s003.pdf]

S3 Table. **Sowing and harvest dates of pearl millet during the study period 2011-2018**. The predicted flowering and harvest dates using the calibrated APSIM-Millet are presented. Values in parentheses correspond to the days after sowing (DAS).

Year	Sowing date	Harvest date	Predicted flowering date	Predicted harvest date [a]
**2011**	20 June	25 September (97)	28 August (69)	22 September (94)
**2012**	12 June	19 September (99)	23 August (72)	18 September (98)
**2013**	2 July	7 October (97)	14 September (74)	9 October (99)
**2014**	1 June	9 September (100)	13 August (73)	7 September (98)
**2015**	9 July	10 October (93)	19 September (72)	14 October (97)
**2016**	15 June	19 September (96)	19 August (65)	13 September (90)
**2017**	14 June	17 September (95)	25 August (72)	19 September (97)
**2018**	24 June	29 September (97)	28 August (65)	23 September (91)
	Average	97 DAS	70 DAS	96 DAS
