# Supplementary figures and images for: Assessing yield stability of pearl millet and rice cropping systems across West Africa using long-term experiments and a modeling approach
Source: PLoS One. 2025 May 27;20(5):e0317170. doi: 10.1371/journal.pone.0317170 (PMC12112412; doi:10.1371/journal.pone.0317170)

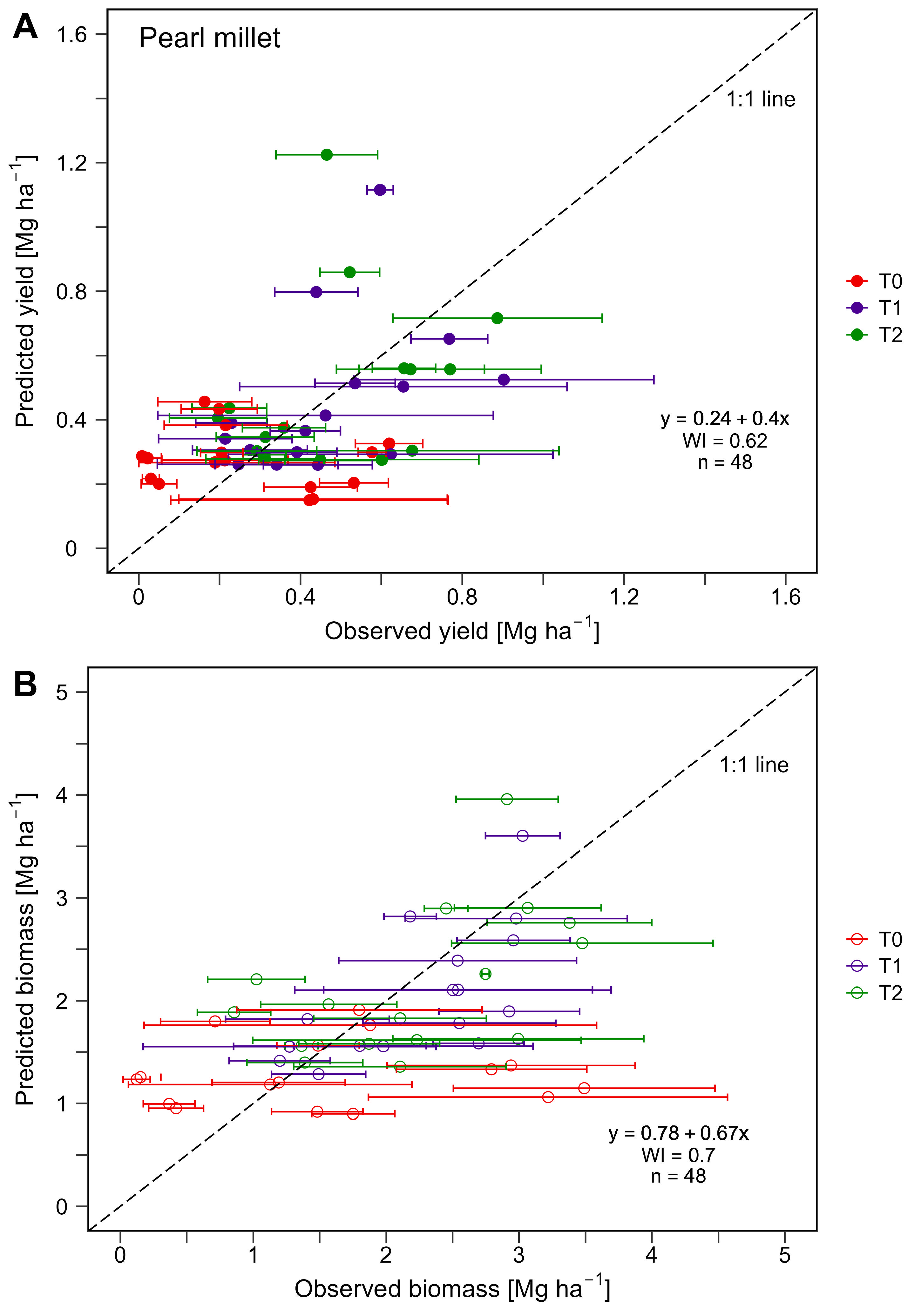

Supplement: S1 Fig — (A) Scatterplots of observed versus predicted grain yield. (B) Scatterplots of observed versus predicted biomass. WI: Willmott’s index of agreement; n: number of data pairs. T0 = control plot; T1 and T2: plots under fertilizer treatment T1 and T2, respectively. (TIF) [file pone.0317170.s004.tif]

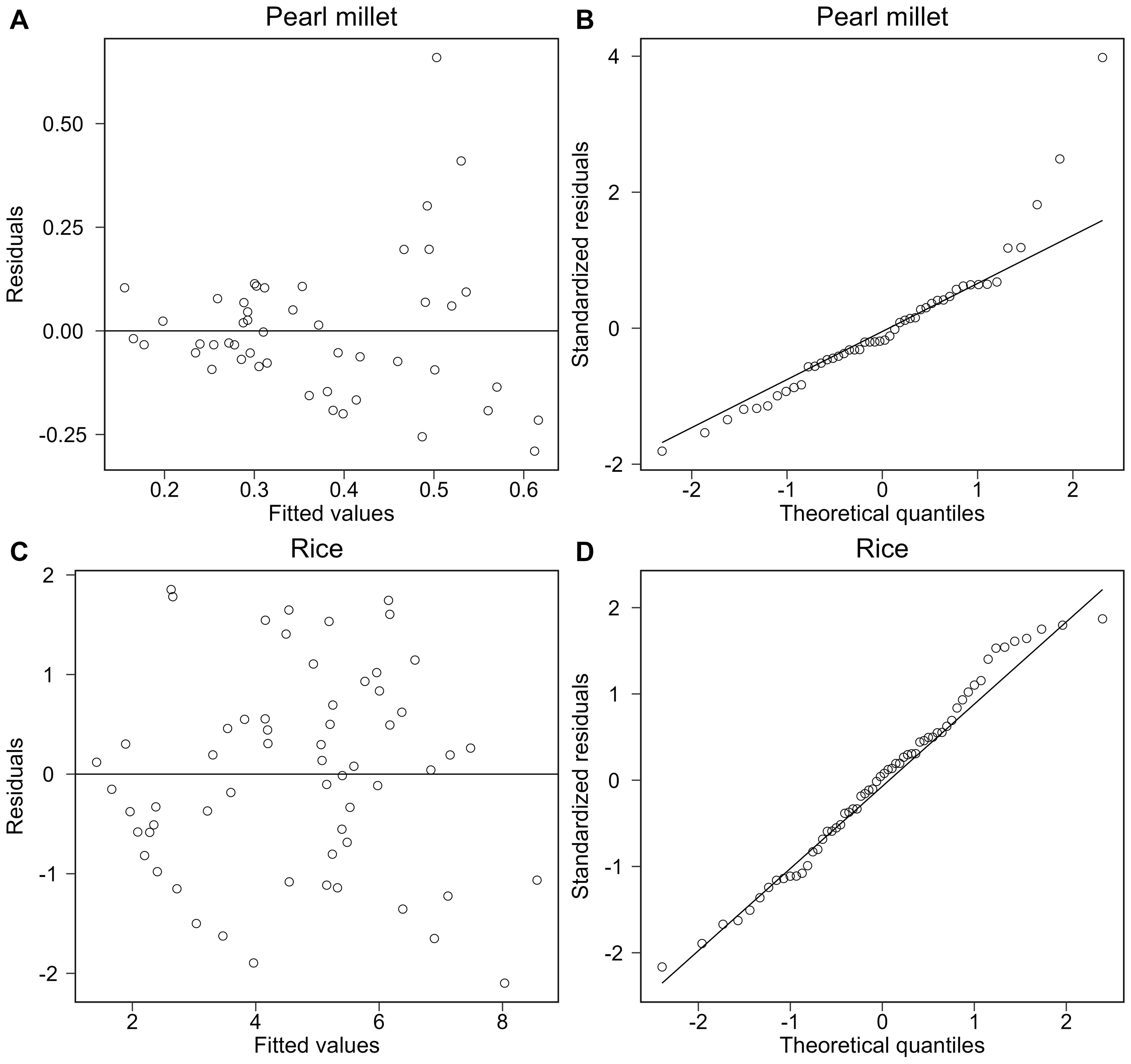

Supplement: S2 Fig — Scatterplots of (A) fitted values vs residuals and (B) theoretical quantiles vs standardized residuals for pearl millet. Scatterplots of (C) fitted values vs residuals and (D) theoretical quantiles vs standardized residuals for rice. Pearl millet data are from plots under crop residue retention. (TIF) [file pone.0317170.s005.tif]

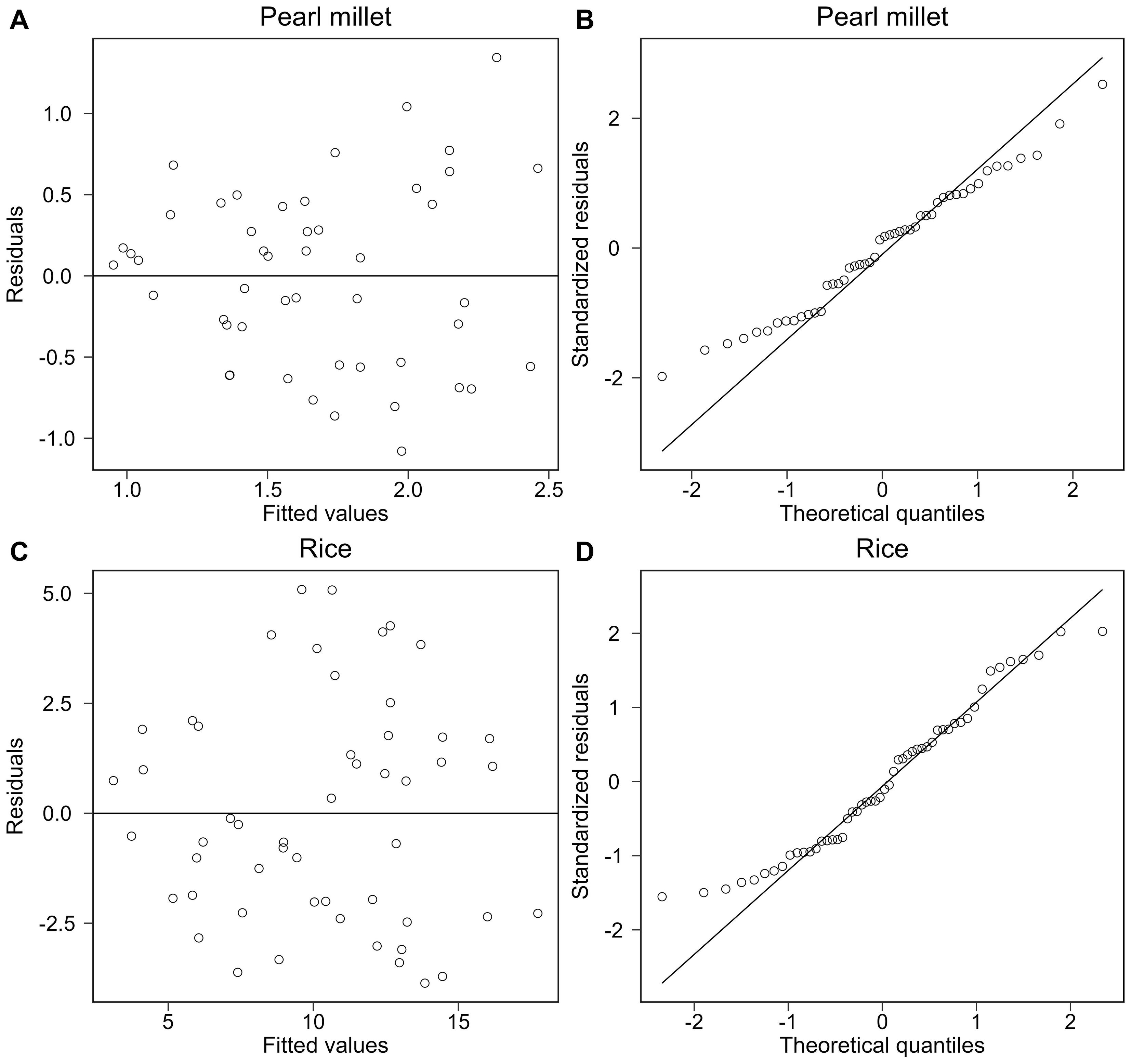

Supplement: S3 Fig — Scatterplots of (A) fitted values vs residuals and (B) theoretical quantiles vs standardized residuals for pearl millet. Scatterplots of (C) fitted values vs residuals and (D) theoretical quantiles vs standardized residuals for rice. Pearl millet data are from plots under crop residue retention. (TIF) [file pone.0317170.s006.tif]

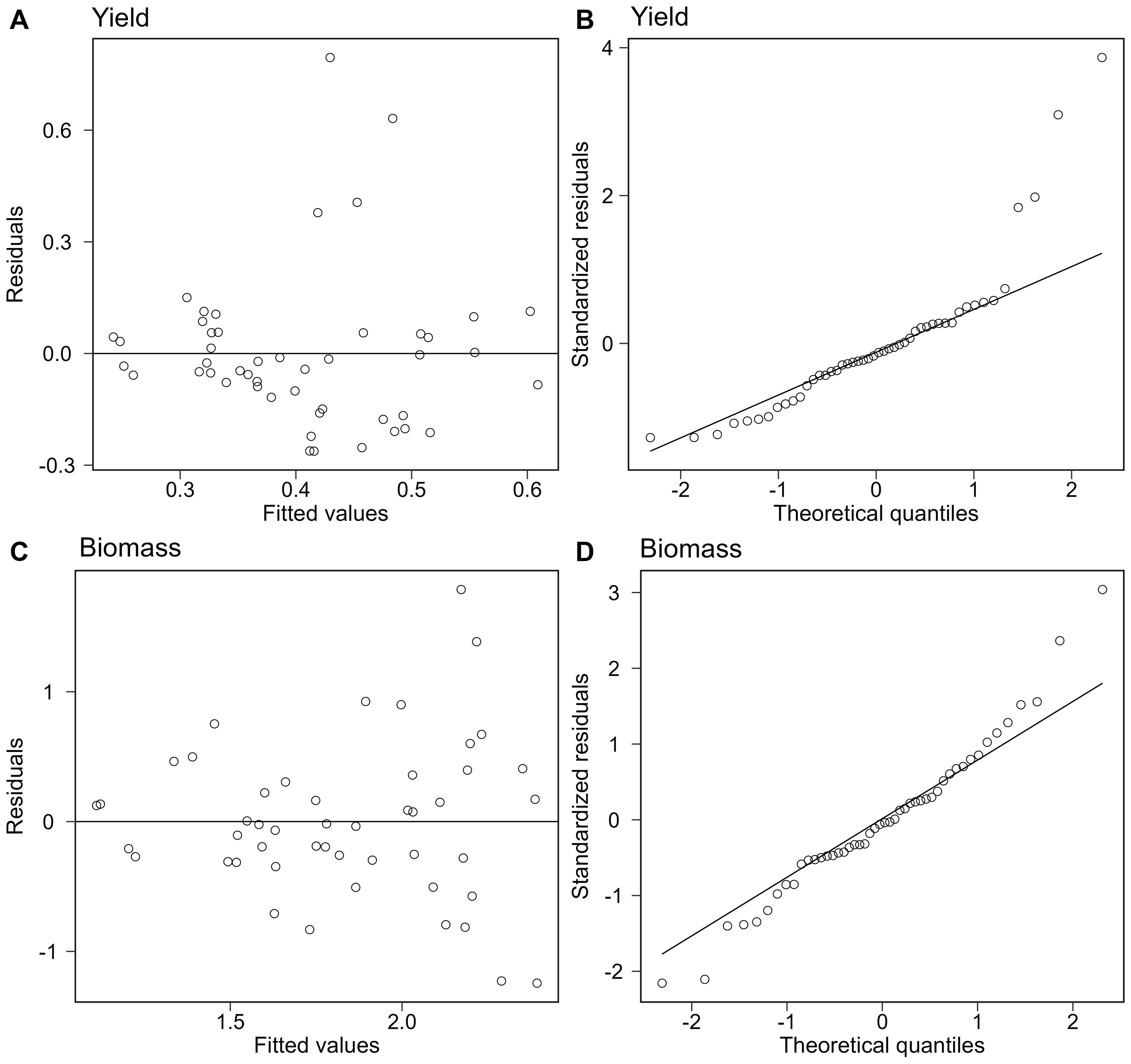

Supplement: S4 Fig — Scatterplots of (A) fitted values vs residuals and (B) theoretical quantiles vs standardized residuals for yield. Scatterplots of (C) fitted values vs residuals and (D) theoretical quantiles vs standardized residuals for biomass. (TIF) [file pone.0317170.s007.tif]

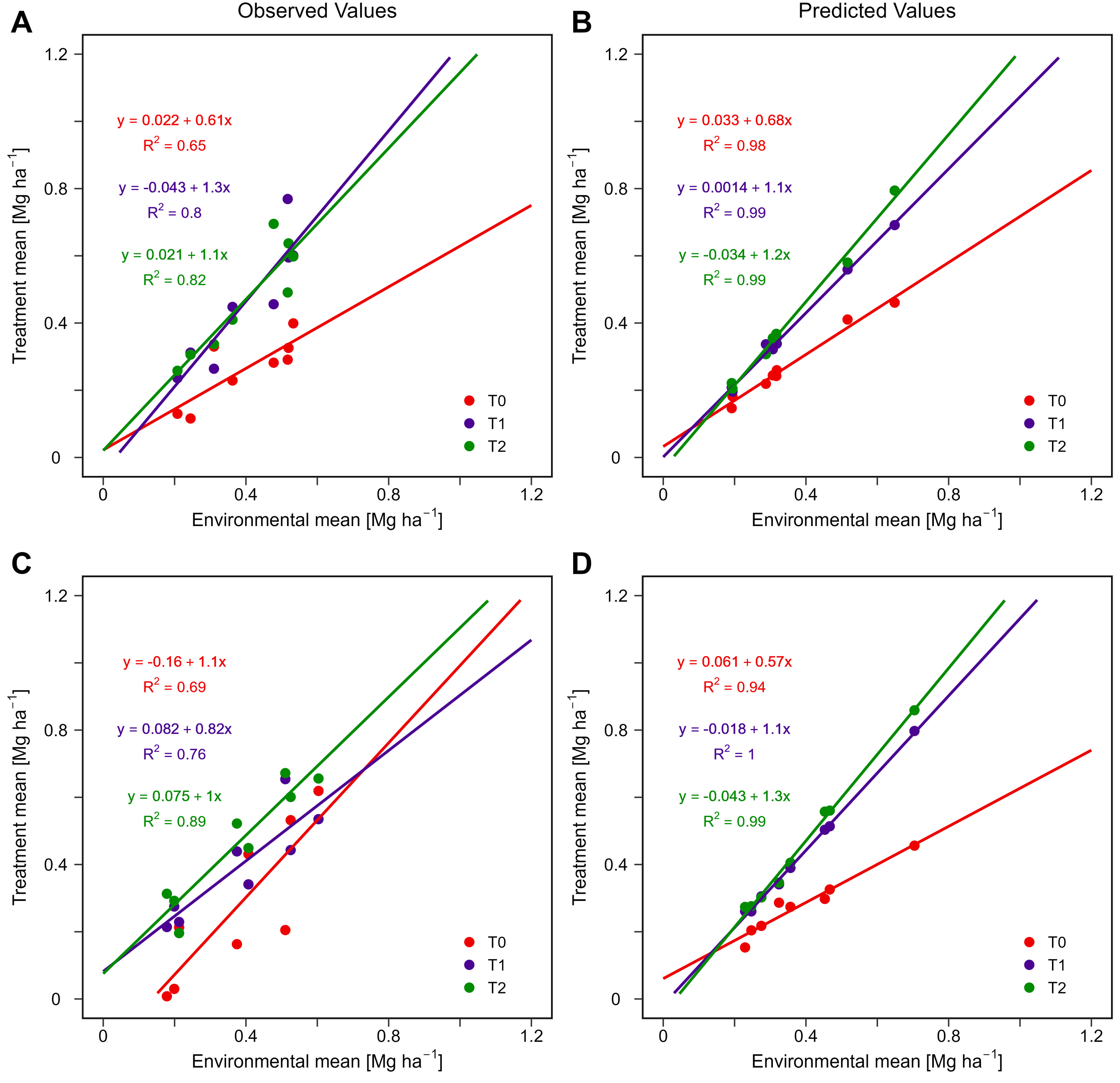

Supplement: S5 Fig — (Top) Plots under crop residue retention: (A) observed and (B) predicted yields. (Bottom) Plots under crop residue removal: (C) observed and (D) predicted yields. Data for plant density PDENS2 (15,000 pockets ha-1) are presented. T0 = control plot; T1 and T2: plots under fertilizer treatment T1 and T2, respectively. (TIF) [file pone.0317170.s008.tif]
